# Untargeted metabolomics screening reveals unique secondary metabolite production from *Alternaria* section *Alternaria*

**DOI:** 10.3389/fmolb.2022.1038299

**Published:** 2022-11-24

**Authors:** Thomas E. Witte, Nicolas Villenueve, Samuel W. Shields, Amanda Sproule, Quinn Eggertson, Natalie E. Kim, Christopher N. Boddy, Jeremy R. Dettman, David P. Overy

**Affiliations:** ^1^ Ottawa Research and Development Centre, Agriculture and Agri-Food Canada, Ottawa, ON, Canada; ^2^ Department of Chemistry and Biomolecular Sciences, University of Ottawa, Ottawa, ON, Canada

**Keywords:** *Alternaria*, fungal plant pathogen, dehydrocurvularin, untargeted metabolomics, mass spectrometry, secondary metabolites, biosynthetic gene cluster, accessory region

## Abstract

*Alternaria* section *Alternaria* is comprised of many species that infect a broad diversity of important crop plants and cause post-harvest spoilage. *Alternaria* section *Alternaria* species, such as *A. alternata* and *A. arborescens*, are prolific producers of secondary metabolites that act as virulence factors of disease and are mycotoxins that accumulate in infected tissues—metabolites that can vary in their spectrum of production between individuals from the same fungal species. Untargeted metabolomics profiling of secondary metabolite production using mass spectrometry is an effective means to detect phenotypic anomalies in secondary metabolism within a species. Secondary metabolite phenotypes from 36 *Alternaria* section *Alternaria* isolates were constructed to observe frequency of production patterns. A clear and unique mass feature pattern was observed for three of the strains that were linked with the production of the dehydrocurvularin family of toxins and associated detoxification products. Examination of corresponding genomes revealed the presence of the dehydrocurvularin biosynthesis gene cluster associated with a sub-telomeric accessory region. A comparison of sequence similarity and occurrences of the dehydrocurvularin biosynthetic gene cluster within Pleosporalean fungi is presented and discussed.

## Introduction

In agriculture, many secondary metabolites produced by plant pathogenic fungi act as virulence factors and mycotoxins involved in the onset of the disease state and/or accumulate in harvested produce and grains, both of which directly impact crop yields and market suitability. The profile of secondary metabolites expressed during disease onset and throughout the pathogen life cycle within a crop can vary between individuals from the same fungal species. One reason for observed within-species variation in secondary metabolism is the presence of lineage-specific accessory chromosomes or subtelomeric accessory regions within the genome that encode for unique secondary metabolite biosynthetic gene clusters. Understanding trends in intraspecific secondary metabolite expression patterns of a pathogen population is therefore of interest to pathologists and regulatory agencies that monitor for the emergence of crop diseases and associated mycotoxins of concern.

Profiling small molecule production using mass spectrometry and untargeted metabolomics tools is an effective means to detect phenotypic anomalies in secondary metabolism within a species (Witte and Overy, 2022). ‘Consensus’ chemical phenotypes representing the breadth in secondary metabolism expressed by a given strain can be generated from extracts of single-spore isolates cultured under axenic conditions on multiple different types of media. Profiling culture extracts using ultra-performance liquid chromatography - high resolution mass spectrometry (UPLC-HRMS) coupled with untargeted metabolomics tools for data processing and analysis are used to designate mass features of interest from chemical phenotype modelling. A mass spectrometry technique called tandem MS involves applying energy to selected mass feature ions (precursor ions) generating molecular fragments (product ions)—when this event is carried out a single time it is designated as MS/MS (or MS^2^). Through MS^2^ experiments, molecular fragmentation patterns of mass features of interest are then compared to online MS^2^ spectral databases for putative metabolite annotation (Dührkop et al., 2015, 2019; Hoffmann et al., 2021). Molecular networking analysis can also be performed to detect deviations in the molecular structures of known secondary metabolite products, indicating if new metabolite analogs are present (Wandy et al., 2018; Nothias et al., 2020).

Combining genomics and untargeted metabolomics is a powerful way to screen populations of fungi for secondary metabolite production resulting from transcriptionally active biosynthetic gene clusters. Recent work in the field has revealed that fast-evolving “accessory” genomic regions, including small “accessory or conditionally dispensable” chromosomes, can often encode isolate-specific secondary metabolite biosynthetic gene clusters which diversify metabolite profiles within a population or species (for review see Witte et al., 2021b). Structural information of observed secondary metabolite products can be correlated with synthase/synthetase motifs from predicted secondary metabolite biosynthetic gene clusters in the genomes, and associations inferred. Recent studies employing this multi-omic approach have proven useful in the discovery of apicidin from *Fusarium poae* (Witte et al., 2021a) and to explain rapidly evolving ergopeptine patterns in *Claviceps purpurea* (Hicks et al., 2021).

*Alternaria* is a speciose, cosmopolitan fungal genus currently divided into 26 sub-generic sections, whose members can be found growing as saprobes, endophytes, pathogens, or something in between (Woudenberg et al., 2013; Woudenberg et al., 2014; Grum-Grzhimaylo et al., 2016). Of particular relevance to the agronomic sector is *Alternaria* section *Alternaria*, which contains many species that infect important crop plants and cause post-harvest spoilage. Commensurate with the broad diversity of plant hosts associated with *Alternaria* section *Alternaria* is the diversity of their associated secondary metabolites, which were the focus of intensive study for at least half a century (Lou et al., 2013). Chemotaxonomy has played an important role in understanding the pathogenic competency of *A. alternata*, the type species of section *Alternaria*. This widespread species is associated with plant disease in a remarkably diverse number of hosts (Rotem 1994), and certain strains have been deemed “*formae speciales*” or “pathotypes” based on their production of phytotoxins required to surmount specific host plant defenses (reviewed in Tsuge et al., 2013; Meena et al., 2017).

Isolates belonging to *A. alternata*, and the closely related *A. arborescens* species complex, are of particular notoriety because they are commonly detected both as plant pathogens and as food spoilage agents producing mycotoxins of concern for consumers. Toxicity data associated with chronic exposure for many *Alternaria* toxins is lacking, however, the genotoxins alternariol and alternariol monomethyl ether have been flagged for further study due to potential deleterious effects on human and animal health (EFSA scientific opinion 2011). Laboratory-based studies of exposure to other *A. alternata* associated toxins, such as tentoxin, tenuazonic acid, altertoxins, altenuene and AAL-toxins have either demonstrated acute toxicity or other harmful effects to animal or cellular models, including teratogenic and fetotoxic effects. Many *Alternaria* metabolites are therefore considered “emerging mycotoxins” of significant regulatory concern, whose effects on plants and plant consumers require further investigation, including in holistic or synergistic multi-toxin contexts.

In the current research study, the aforementioned untargeted metabolomics profiling techniques (Witte and Overy, 2022) were leveraged to characterize and compare UPLC-HRMS secondary metabolite profiles of representative *Alternaria* extracts derived from axenic cultures on differing media. Untargeted chemical phenotypes from 36 *Alternaria* isolates were constructed by converting preprocessed metabolomics data into simple binary matrices representing mass feature presence/absences per media tested which were in turn combined to observe frequency of production patterns. Binary matrices were used to simplify interpretation of secondary metabolite phenotypes by eliminating mass feature intensity variation which may arise from extract concentration variability or fungal competence levels (Witte and Overy, 2022). A clear and unique mass feature pattern was observed for three of the strains that were linked with the production of the dehydrocurvularin family of toxins and associated detoxification products. Examination of corresponding genomes revealed the presence of the dehydrocurvularin biosynthesis gene cluster (BGC) associated with a sub-telomeric accessory region. Comparison of the observed dehydrocurvularin BGC within Pleosporalean fungi is presented and discussed in terms of the potential of horizontal gene transfer between plant pathogens.

## Materials and methods

### Fungal strains

A total of 36 *Alternaria* strains identified to *Alternaria* section *Alternari*a were selected for study. For strains not previously identified to species, section-specific diagnostic markers *ASA-10* and *ASA-19*, and the commonly used marker RNA polymerase II second largest subunit (*rpb2*), were sequenced for species determination at greater taxonomic resolution. Sequences were aligned with a representative set of strains and phylogenetic analyses were used to place strains within species or lineages (data not shown). Barcode DNA extraction, polymerase chain reaction (PCR), Sanger sequencing, representative strains, and phylogenetic analyses were as described in Dettman & Eggertson (2022).

### Media and growth conditions

The four medium types used for axenic growth of fungal strains were CYS80, DRYES, MMK2, and ZM2 (media formulations are provided in the Supplementary Section 1.1 Media Formulations). For metabolomic profiling, all strains were 3-point inoculated onto plates of the four different media, with four plate replicates per medium. Inoculated plates were incubated for 14 days under conditions of 25°C and a light/dark cycle of 8/16 h. Additional media control plates (non-inoculated) were included.

### Metabolite extraction

Nine agar plugs (5 mm diameter) were harvested from each fungal replicate into distinct scintillation vials and frozen at −20°C until they were able to undergo chemical extraction. Extractions were performed with 10 ml ethyl acetate (EtOAc) per vial; each vial was shaken on a rotary shaker for 3 h (at 160 RPM). Resulting solvent extracts were transferred to clean scintillation vials and evaporated until dry using a GeneVac (SP Scientific EZ-2 Elite) and stored at −20°C prior to UPLC-HRMS analysis. Details of chemicals/solvents and associated manufacturers are provided in (Supplementary Section 1.2 Chemical and Solvent List).

### UPLC-HRMS analyses

Dried metabolite extracts were suspended in 1 ml of methanol (MeOH) and analyzed on a Thermo Ultimate 3000 UPLC coupled to a Thermo LTQ Orbitrap XL high resolution mass spectrometer and a Thermo Dionex Ulitmate 3,000 Diode array detector (190–800 nm). Chromatography was performed using a Phenomenex C_18_ Kinetex column (50 mm × 2.1 mm ID, 1.7 μm) with a flow rate of 0.35 ml/min over 10-min run, using a gradient of acetonitrile (ACN) (+0.1% formic acid) and water (H_2_O) (+0.1% formic acid): starting at 5% ACN and increased to 95% ACN by 4.5 min; held at 95% ACN until 8 min; returned to 5% ACN by 9 min; and then held at 5% ACN to 10 min to equilibrate the column to starting conditions. The HRMS was operated in electro-spray ionization mode (ESI^+^), monitoring a range of 100–2,000 *m/z*). The ESI used a capillary temp of 320°C, a sheath gas flow of 40, an auxiliary gas flow of 5, a sweep gas flow of 2, a source voltage of 4kV, a capillary voltage of 35 V, and a tube lens of 100 V.

MS^n^ fragmentation was performed in high resolution on select ions in subsequent experiments using CID at 35 NCE. MassWorks™ software (v5.0.0, Cerno Bioscience) was used to improve spectral accuracy and confirm the molecular formulas of annotated ions. The sCLIPS searches were performed in dynamic analysis mode with elements C, H, N, and O allowances set at minimum 1 and maximum 100. Charge was specified as 1 and mass tolerance set to 5 ppm.

### NanoLC-HRMS/MS analyses

Chromatographic separation of metabolites was performed on a Proxeon EASY nLC II System (Thermo Fisher Scientific) equipped with a Thermo Scientific™ Acclaim™ PepMap™ RSLC C18 column (15 cm × 75 μm ID, 3 μm, 100 Å) employing a 60 min, H_2_O/ACN (0.1% formic acid) gradient at a flow rate of 0.25 μL/min. Compounds were separated using a linear gradient from 10 to 100% ACN for 45 min, followed by washing 5 min at 100% ACN, then using a gradient from 100 to 10% ACN for 5 min and washing for 5 min at 100% H_2_O. Eluted compounds were analyzed on a Thermo Q-Exactive Plus HRMS using positive electrospray ionization (ESI) at an ion source temperature of 250°C and an ionspray (Thermo Scientific™ EASY spray) voltage of 2.1 kV. The FTMS scan type was full MS/data dependent (dd)-MS2 with the following parameters: a resolution of 70,000, an auto gain control target under 3.0^6^, a maximum isolation time of 100 ms, and an *m/z* range of 100–1,500. The parameters of the dd-MS2 scan were as follows: a resolution of 17,500, an auto gain control target under 1.0^5^, a maximum isolation time of 100 ms, a loop count of top 10 peaks, an isolation window of *m/z* 2, a normalized collision energy of 35 and dynamic exclusion duration of 10 s.

### Metabolomics: Data preprocessing, curation, and visualization

UPLC-HRMS raw data files were converted to centroid format using Proteowizard MSConvert (opensource software: https://proteowizard.sourceforge.io/) to convert the datafiles into centroided. mzML format prior to preprocessing using MZmine 2.51 (Pluskal et al., 2010). Raw data files included MeOH blanks run after every sixth sample, and all were carefully examined to determine a minimum noise level threshold for data analysis. Mass feature detection was performed using the *centroid mass detection* algorithm using a noise level cut off of 9.0 × 10^4^. ADAP chromatogram building was performed using a minimum groups size of 5 scans, a group intensity threshold of 1.0 × 10^5^, a minimum highest intensity set to 1.0 × 10^7^, and the *m/z* tolerance set to 10 ppm. Chromatogram deconvolution was performed using the local minimum module, with a chromatographic threshold set to 35%, a search minimum retention time range 0.05 min, a minimum relative height at 15.0%, a minimum absolute height at 5.0 × 10^6^, and a minimum ratio of peak top/edge at 1.2, with a peak duration max of 2.00 min. Monotonic shape isotope grouping was performed using a *m/z* tolerance of 8.0 ppm, a retention time tolerance of 0.08 min, and a maximum charge of 1. Mass feature peak alignment was performed using the join aligner with the *m/z* tolerance set to 10.0 ppm (with a weight value set to 2) and the retention time tolerance set to 0.1 min (with a weight value set to 1). Finally, the multithreaded peakfinder *gap-filling* algorithm was applied to the dataset with an intensity tolerance of 70%, the *m/z* tolerance parameter specified as 10.0 ppm and the retention time tolerance set to 0.08 min. The data was then normalized to the total ion current prior to export. Selection of the utilized parameters was informed by inspection of the raw spectra, and accounting for the variance in the caffeine standard (included in the extraction solvent) and reserpine calibration standard which was run prior to each batch of samples.

A data matrix containing mass feature *m/z*, retention time, and normalized height was exported from MZmine and data curation, binary conversion and mass feature frequency phenotyping was performed in the R environment using in-house scripts according to Witte and Overy (2022). Mass features associated with media components and UPLC-HRMS system contaminants were removed. Data reduction was performed using Pearson correlation analysis over a sliding elution time window by grouping mass features if their intensities correlated across samples (correlation coefficients threshold set to 0.8 with a maximum p-value of 0.05) and if they eluted from the column within 0.02 min of each other. Mass feature peak height binary conversion was then performed and sample replication was compressed into a single mass feature value using a filtering step, where mass features were assigned a ‘0’ if they occurred in less than 3 of the 4 replicates (ie., <0.75). The data matrix was further reduced by summing mass feature values across the 4 media tested to form a ‘pseudo-binary’ matrix of detection frequencies for each feature. For the generation of the chemical phenotype heatmap, row and column clustering was calculated using “ward.D2” clustering of Euclidean distances. The heatmap was generated using the “Complexheatmap” R package (Gu et al., 2016).

### MS^2^ molecular network analysis

One replicate extract of *Alternaria arborescens* strain DET2035 grown on CYS80 media was selected as a representative sample and submitted to obtain increased chromatographic resolution of metabolite components and untargeted HRMS/MS analysis for use with molecular ion network modelling. Data preprocessing was performed using MZMine2 pre-release version 2.37.1corr17.7 utilizing the *Ion Identity Networking* (IIN) module (Schmid et al., 2020). MS1 and MS2 mass detection noise levels were set to 1.0 × 10^5^ and 1.0 × 10^2^ respectively, all *m/z* tolerances were set to 10 ppm, minimum highest intensity for chromatograph building and deconvolution was set to 1.0 × 10^7^, and maximum peak duration range was set to 8 min. Ion identities (adducts or in-source fragments) were determined by first grouping features using the *Metacorrelate* module with the following parameters: RT tolerance set at 0.08 min, minimum height at 1.0 × 10^5^, noise level at 1.0 × 10^4^, and with correlation grouping parameters set to 5 minimum data points, 2 minimum data points on edge and Pearson correlation minimum feature shape correlation at 85% (minimum total correlation not checked). The IIN module in MZmine was then used to annotate adducts and simple fragment ions within correlation groupings on the assumption that correlated groupings represent fragments and adducts from the same parent ion. The parameters for this module were as follows: the *m/z* tolerance was set to 10 ppm, with a minimum height of 1.0 × 10^7^, and a simple annotation library was used containing only [M+H]^+^ [M+Na]^+^, [M+NH_4_]^+^, and [M-H_2_O]^+^ as potential adducts and modifications, with a maximum charge of 1 and with a maximum of 2 molecules/cluster. Features were filtered from the data if they lacked annotation and/or MS^2^ scans.

Preprocessed features were networked using feature-based molecular network analysis as part of the GNPS FBMN online workflow (version release_28.2) (Nothias et al., 2020). MS^2^ spectra (nodes) were linked to form clusters in the network if they had at least 6 matched fragment ions and a cosine score greater than 0.75, or if they had annotated ion identities within correlation groupings. Default settings were used for the FBMN workflow with the following exceptions: Network TopK was set to 20, and Maximum Connected Component Size was set to 0 (unlimited). The resulting analysis was imported and visualized in Cytoscape v 3.9.1. Nodes were coloured based on ion identity. To predict the presence of shared mass motifs in the MS2 scans, the dataset was analyzed using MS2LDA (release_31) through the GNPS web interface (Wandy et al., 2018). The resulting mass motifs were imported into cytoscape and merged with FBMN data to visualize motif presence patterns. The network was then reduced by collapsing nodes linked as adducts/fragments either *via* the IIN module or by manual annotation of adducts and fragments which the IIN module had missed (by mathematical relationships and extract ion chromatogram peak shape comparison in Thermo Xcalibur).

### Mass feature annotation

UPLC-HRMS/MS and nanoLC-HRMS/MS MS^2^ scans were compared to freely available libraries of experimentally derived MS^2^ data *via* the GNPS online interface (version 28.2). Matches were made if they had cosine similarity scores over 0.7, and at least 6 matched fragment ions. All libraries available *via* GNPS were selected by default, including the Massbank of North America and the GNPS natural products library. In addition to the above, CSI Finger-ID version 5.5.5 was utilized to interpret MS^2^ scans of the features of interest for *in silico-*based chemical formula and structural prediction (Dührkop et al., 2015, 2019; Hoffmann et al., 2021). CSI Finger-ID performs high-resolution isotope pattern analysis in combination with fragmentation tree analysis to assist in structure elucidation, comparing MS^2^ data to chemical structures harvested from online databases (such as PubChem, ChemSpider, KEGG, MetaCyc, MINE, CHEBI, UNPD, etc.). Predictions resulting from *in silico* analyses were interpreted with reference to a previously-compiled database of 300+ secondary metabolites identified and associated with *Alternaria* species in scientific literature, to filter out suggestions inappropriate to the taxa under study.

For features not annotated by either experimental or *in silico* derived comparisons listed above, identities were predicted through comparison of the pseudomolecular ion masses to the above-mentioned literature-based *Alternaria* secondary metabolite database, and MS^2^ spectra/mass motifs were manually examined to support the resulting annotations.

### Whole-genome sequencing and assembly

Strains were grown on potato dextrose agar (PDA) plates at 25°C for 14 days. Genomic DNA extraction and short-read sequencing were performed as described in Dettman & Eggertson (2021). In brief, genomic DNA samples were sheared to approximately 350 bp fragment lengths using a Covaris M220 sonicator (Covaris, Massachusetts), PCR-free libraries were constructed with a NxSeq AmpFREE Low DNA Library Kit (Lucigen, Wisconsin) and sequenced with paired-end reads (2 × 150bp) on a Illumina NextSeq500 instrument (Illumina, California) using a NextSeq High Output Reagent Kit. For long-read sequencing with a MinION device (Oxford Nanopore Technologies, UK), libraries were prepared from genomic DNA using kit SQK-LSK109. Libraries were run for 48 h on a FLO-MIN106 flow cell following the manufacturer’s recommended protocols, with a resulting estimated read N50 of 13.8 Kb. Basecalling to fastq format was performed with GUPPY (4.2.2, https://nanoporetech.com/community) under default settings.

Genome assembly from short-read data was performed as described in Dettman & Eggertson (2021). In brief, raw reads were clipped of adapters and quality trimmed with TRIMMOMATIC (0.38, Bolger et al., 2014) and assembled with SPADES (3.12, Bankevich et al., 2012). Contigs were removed if they were less than 500 bp in length, or if BLASTn searches suggested they were bacterial or mitochondrial in nature. To estimate the completeness of genomes, the BUSCO (benchmarking universal single-copy orthologs; 4.0.6, Simão et al., 2015; Seppey et al., 2019) metric was determined for nucleotide-level assemblies using the *ascomycota_odb10* ortholog dataset as reference.

Genome assembly from long-read data was performed with CANU (2.1.1; Koren et al., 2017) with genomeSize = 34 m, correctedErrorRate = 0.154, and minOverlapLength = 1,000 (all other settings were default). The assembly was polished by signal-level analyses using NANOPOLISH (0.13.2; https://github.com/jts/nanopolish) (Loman et al., 2015). Four successive rounds of error-correction were then performed with short-read data using PILON (v1.23; Walker et al., 2014). Chromosome 8 was identified *via* whole chromosome alignments against the *A. solani* NL03003 genome assembly, performed with progressiveMAUVE (2.4; Darling et al., 2004).

### Gene prediction and annotation

Gene prediction and annotation were performed using FUNANNOTATE (1.8.7; https://github.com/nextgenusfs/funannotate; Palmer and Stajich., 2020) with methods described in Dettman and Eggertson, 2021. A modification made here was that evidence from SNAP and CodingQuarry were excluded during the EvidenceModeler step. Genome-wide gene predictions were run through antiSMASH (6.0, Blin et al., 2021) to identify secondary metabolite gene clusters. Repetitive and transposable elements were identified using the TransposableELMT wrapper script (1.0.0; https://github.com/PlantDr430/TransposableELMT) described in Wyka et al., 2021.

### Search for dehydrocurvularin BGC homologs in Pleosporalean fungi

Searches of NCBI databases included full gene and coding sequence queries against the “nt” database using BLASTn, and protein sequence queries against the “nr” database using BLASTp. Additional searches against “refseq_genomes”, “refseq_representative_genomes”, and “wgs” databases did not expand the taxonomic diversity of hit subjects so the results are not presented. Next, genomes of Pleosporalean fungi that were publicly available from sequence repositories (e.g. NCBI, JGI Mycocosm) were downloaded (accessed 8 December 2021) and a custom, local BLAST database was created. The local Pleosporalean database was queried with BLASTn searches with a relaxed threshold of 70% minimum similarity to allow detection of potentially highly divergent genes. The dehydrocurvularin BGC was considered present in a genome if both of the polyketide synthase genes had full length hits.

## Results

### Taxonomy of *Alternaria* strains

Each of the *Alternaria* strains used in this study were micro-morphologically examined and found to produce branched chains of transverse and longitudinally septate, melanized conidia with tapering beaks. These micro-morphological observations, along with characteristics of culture growth, were consistent with taxonomic descriptions of species belonging to the small-spored *Alternaria* section *Alternaria*. Twelve strains were previously identified to species/lineage in Dettman & Eggertson (2021), Dettman & Eggertson (2022). For the remaining 24 strains, we sequenced three informative molecular markers (*ASA-10*, *ASA-19*, and *rpb2*) and assigned strains to species/lineage with phylogenetic analyses (data not shown) using the approach described in Dettman & Eggertson (2022). Overall, the majority of the 36 strains belonged to *A. alternata* (*n* = 23) or the *A. arborescens* species complex (*n* = 11), with one *A. longipes* strain, and one strain that could not be placed in a known lineage. An example of species determination *via* phylogenetic analysis for 15 representative strains is provided in Supplementary Figure S3).

### Untargeted metabolomics profiling and detection of dehydrocurvularin-like mass features

**FIGURE 1:**
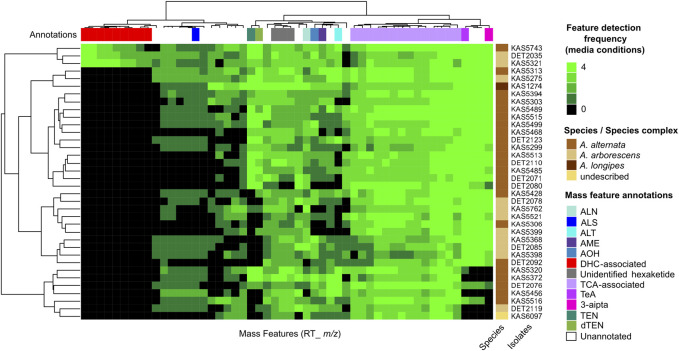
Untargeted metabolomics analysis of 36 *Alternaria* isolates, mainly from *A. alternata* and *A. arborescens*. Columns represent mass features, with heatmap values representing detection frequencies across four media conditions. Hierarchical cluster analysis was used to group isolates by the similarity of their mass feature detection frequencies (row dendrogram), and mass features by the similarity of their frequencies between isolates (column dendrogram). Annotations in the top colour bar are interpretations based on representative mass feature annotations: ALN, altersetin; ALS, altenusin; ALT, altenuene; AME, alternariol monomethyl ether; AOH, alternariol; DHC-associated, dehydrocurvularin and associated mass features annotated in this study; TCA, tricycloalternarene-associated mass features; TeA, tenuazonic acid-associated mass features; 3-aipta, 3-acetyl-5-isopropyltetramic acid; TEN, tentoxin; dTEN, dihydrotentoxin.

Trends in the occurrence and frequency of secondary metabolite production between *Alternaria* strains were visualized using a consensus metabolomic phenotype heatmap (Figure 1). The phenotype heatmap was derived from UPLC-HRMS profiling of culture extracts from four media conditions and following data reduction steps, consisted of 52 representative mass features. Mass features with *m/z* matching the exact mass (within 5 ppm) of monoisotopic adducts of known *Alternaria* mycotoxins altenuene, altenusin, alternariol, alternariol monomethyl ether, altersetin, tricycloalternarene 2b, tenuazonic acid, 3-aipta (an analog of tenuazonic acid), tentoxin and dihydrotentoxin were annotated by analysis of MS^2^ fragmentation patterns using *in silico* and MS^2^-database derived comparisons. There were numerous mass features which shared detection frequencies and similar predicted chemical formulas to tricycloalternarene 2b, indicating they are likely related terpenoid compounds, most likely in the bi- or tri-cycloalternarene family, and were annotated as “TCA-associated”. Additionally, there were a number of unannotated mass features which did not share *m/z* with known *Alternaria* secondary metabolites. Among the unannotated signals, three isomeric mass features were detected from extracts of all isolates profiled, and were predicted to share a chemical formula of C_12_H_10_O_5_ (8 unsaturations predicted). We tentatively annotated these three signals as “unidentified hexaketide”.

Detection patterns of the annotated mass features showed interesting patterns of variance between strains and between the secondary metabolites themselves: some were detected in all or nearly all 36 strains profiled, including altersetin (*n* = 34), alternariol (*n* = 32), alternariol monomethyl ether (*n* = 31), tenuazonic acid (*n* = 32), 3-aipta (*n* = 30) and the TCA-associated mass features (*n* = 36). Others were less frequently detected but were still produced by a majority of isolates, including tentoxin (*n* = 23), dihydrotentoxin (*n* = 26), altenuene (*n* = 26) and altenusin (*n* = 24). Hierarchical clustering of mass feature detection frequency patterns between isolates did not correlate with species or species complex identifications.

Interestingly, analysis of the chemical phenotypes indicated isolates DET2035, KAS5321 and KAS5743 produced a group of mass features which were not detected from any of the other isolates (Figure 1, red colour bar). This group of features included exact *m/z* matches (<5 ppm) to known masses from adducts of the ‘dehydrocurvularin’ family of molecules, namely curvularin, dehydrocurvularin, hydroxycurvularin, methoxycurvularin, sumalarin C, and cyclothiocurvularin (Table 1). Confirmation of dehydrocurvularin production was obtained through purification of the molecule from a scaled-up fermentation of DET2035 on solid CYS80 media, followed by comparison of 1D and 2D NMR and HRMS/MS experimental results to literature data (Hassan, 2007; Aly et al., 2010). A full accounting of the detailed purification and structural elucidation of dehydrocurvularin is provided in the Supplementary Section 4.0–4.10. A MS^2^ mass motif associated with the fragmentation of the dehydrocurvularin [M+H]^+^ ion was derived from MS/MS experimental data using both the MS2LDA mass motif analysis tool and by manual examination of MS^2^ spectra (Table 1—red font). Application of the derived dehydrocurvularin MS^2^ mass motif was essential for the association and interpretation of various other dehydrocurvularin-associated mass features.

**TABLE 1:** Relevant observed [M+H]^+^, associated MS^2^ fragment ions *m/z* and UV maxima from dehydrocurvularin-associated metabolites annotated in this study by UPLC-HRMS/MS-DAD.

Metabolite	Observed [M+H]^+^	Observed MS² fragment ions (*m/z*)	UV maxima (nm)
curvularin	293.13779	275, 247, 205, 195, 193, 177, 169, 167, 125, 123, 95, 81, 55	200, 255, 290, 300, 335 (shld)
dehydrocurvularin	291.12239	273, 245, 205, 195, 193, 177, 167, 123, 95, 81, 55	205, 230, 295, 335 (shld)
11-hydroxycurvularin	309.13217	291, 273, 245, 193, 177, 169, 123, 95, 81, 55	220, 275, 305 (shld)
11-methoxycurvularin	323.14826	305, 291, 273, 245, 205, 195, 193, 177, 167, 123, 95, 81, 55	200, 220, 240, 265, 300 (shld)
sumalarin C	413.12540	291, 273, 245, 203, 195, 193, 177, 167, 123, 105, 95, 81, 55	200, 220, 275, 302 (shld)

A set of mass features containing the dehydrocurvularin MS^2^ mass motif and predicted to contain a sulphur atom (based upon isotopic ratios) were associated with the ‘sumalarins’, which is consistent with the hypothesis that structural differences in sumalarin analogs are located on the C11 thioether-linked functional group. The sumalarin C MS^2^ spectrum differed slightly from the dehydrocurvularin MS^2^ motif as it contained a fragment ion with a *m/z* of 203, and the 205 *m/z* ion was reduced to very low levels (rationalization of the sumalarin C annotation based on MS/MS experiments is provided in Supplementary Section 5.0–5.4. Curvularin fragmentation was also generally consistent with dehydrocurvularin MS^2^ motif, however some fragment ions were 2 mass units greater, consistent with an alkane bond between C10 and C11 as found in curvularin. Similarly, 11-hydroxycurvularin and 11-methoxycurvularin-associated mass fragment features had minor variations on the dehydrocurvularin MS^2^ motif. Our MS^2^ fragmentation motif findings agree with literature precedents of fragment ions with a *m/z* of 273, 195, 167, and 123 reported from dehydrocurvularin (Hassan, 2007; Jiang et al., 2008). Additionally, the observed UV maxima from dehydrocurvularin, 11-hydroxycurvularin, 11-methoxycurvularin and sumalarin C are consistent with reported UV absorbance spectra from the literature (Kobayashi et al., 1988; Lai et al., 1989; Meng et al., 2013).

### Feature-based molecular network analysis of *A. arborescens* curvularins

**FIGURE 2:**
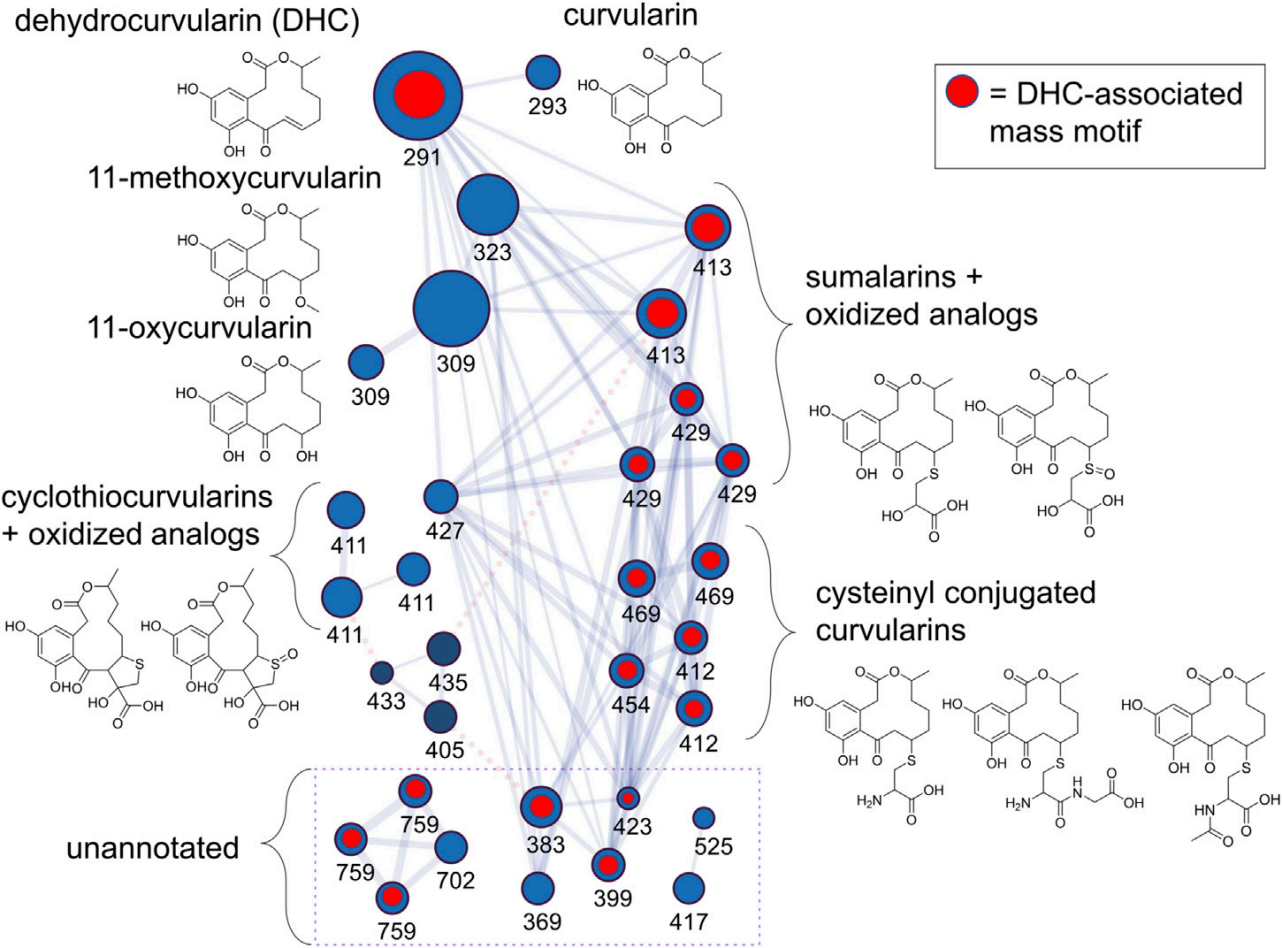
Dehydrocurvularins, sumalarins and related molecules annotated by analysis of MS^2^ fragmentation data from an extract of isolate DET2035. Nodes (circles) represent mass features, where size is proportional to the ion precursor intensity, and are labeled by their nominal mass (see Table 2 for exact masses and annotation details). Nodes are linked with a blue line if their MS^2^ spectra share a minimum of 6 fragment ions, and a cosine score >0.75. Nodes linked with a dotted red line represent putative adducts of the same molecule (linked *via* the ion identity networking module of MZMine2, see methods), and were not collapsed in the network in order to show connectivity between the cyclothiocurvularins and the other signals in the cluster. Blue nodes represent [M+H]^+^ adducts and dark blue nodes represent [M+Na]^+^ adducts. Structures drawn are hypothetical annotations based on exact mass and MS^2^-analysis. The network is derived from the GNPS feature based molecular network analysis workflow incorporating a mix of manually annotated ion identities, and MZMine2-derived ion identities.

Next, we processed an extract of a dehydrocurvularin-producing strain (DET2035) using a nanoLC-HRMS/MS in order to increase our chromatographic resolution and build an extensive library of MS^2^ scans from mass features potentially representing additional dehydrocurvularin-related molecules. Using the feature-based molecular networking workflow as part of the GNPS online suite of tools for MS^2^ analysis, we built a molecular network of dehydrocurvularin-associated mass features (Figure 2). Features matching dehydrocurvularin, curvularin, and sumalarin C were annotated by comparison to experimentally derived MS^2^ data from the GNPS and Massbank spectral libraries as part of the GNPS workflow. Additionally, mass features matching 11-hydroxycurvularin, 11-methoxycurvularin, cyclothiocurvularin, cyclosulfoxicurvularin and sumalarin A were annotated *via in silico* analysis of MS^2^ fragmentation patterns using SIRIUS/CSI-FingerID (relevant GNPS and CSI-FingerID annotation scores are provided in Supplementary Section 6.1, 6.2. Lastly, some mass features had no match to predicted structural fragmentation patterns, usually because the structures were not present in online libraries. Mass features associated with this category included dehydrocurvularin-S-cysteine, dehydrocurvularin-S-cysteinylglycine, dehydrocurvularin-S-(N)-acetyl-cysteine, cyclosulfoxicurvularin and sulfoxicurvularin, which were tentatively annotated using predicted molecular formula based on *m/z* and isotopic ratios as well as by comparison to dehydrocurvularin-associated MS^2^ fragmentation patterns.

The dehydrocurvularin MS^2^ motif was also detected in a number of mass features which were predicted to have nitrogen in their chemical formulas (Table 2), suggesting the production of dehydrocurvularin analogs with cysteine or modified cysteine-like functional groups at the C11 position (Figure 2). Additionally, at least eight mass features were detected at low intensities which either grouped with the molecular network and/or had MS^2^ fragmentation patterns matching the dehydrocurvularin MS^2^ motif. These eight features ranged in *m/z* from 369.13735 to 759.27629, and had unique peak shapes/retention times in our chromatography, suggesting the masses in the 700 + Da region were not a result of dimer adducts formed during ionization. We did not structurally elucidate these features and note that they are present only at relatively low intensities. Additionally, chlorinated forms of dehydrocurvularin, as have been reported from *Alternaria* and *Cochliobolus* isolates (Ghisalberti and Rowland 1993; Bashyal et al., 2017), were not observed in any extract in this study.

**TABLE 2:** Isolate-specific mass features linked to dehydrocurvularin (DHC) detection in this study. All mass features are [M+H]^+^ adducts, with observed *m/z* detected within 5 ppm accuracy to the exact mass of the predicted chemical formula. Retention times are from the nanoLC-HRMS based analysis of an extract from DET2035.

Annotation	Formula [M+H]^+^	Observed [M+H]^+^	RT (min)
dehydrocurvularin^b^	C_16_H_19_O_5_	291.12243	34.21
curvularin^b^	C_16_H_21_O_5_	293.13798	34.59
11-hydroxycurvularin^a^	C_16_H_21_O_6_	309.13160	31.35
11-hydroxycurvularin^a^	C_16_H_21_O_6_	309.13164	28.29
11-methoxycurvularin^a^	C_17_H_23_O_6_	323.14789	32.17
	C_18_H_25_O_6_S	369.13735	31.9
	C_18_H_23_O_7_S	383.11491	31.97
	C_18_H_23_O_8_S	399.10959	30.33
cyclothiocurvularin A/B^a^	C_19_H_23_O_8_S	411.10907	33.23
cyclothiocurvularin A/B^a^	C_19_H_23_O_8_S	411.10925	31.94
DHC-S-cysteine	C_19_H_26_O_7_NS	412.14120	26.073
DHC-S-cysteine	C_19_H_26_O_7_NS	412.14126	26.53
sumalarin C^b^	C_19_H_25_O_8_S	413.12552	30.43
sumalarin C^b^	C_19_H_25_O_8_S	413.12551	30.64
	C_22_H_25_O_8_S	417.15388	31.51
	C_21_H_27_O_9_	423.16700	29.66
cyclosulfoxicurvularin^a^	C_19_H_23_O_9_S	427.10521	26.9
sumalarin A^a^	C_20_H_27_O_8_S	427.14210	32.66
Sulfoxicurvularin	C_19_H_25_O_9_S	429.12150	27.41
Sulfoxicurvularin	C_19_H_25_O_9_S	429.12100	27.72
Sulfoxicurvularin	C_19_H_25_O_9_S	429.12130	27.2
DHC-S-(N)-acetylcysteine	C_21_H_28_O_8_NS	454.15440	30.66
DHC-S-cysteinylglycine	C_21_H_28_O_8_N_2_S	469.16457	24.92
DHC-S-cysteinylglycine	C_21_H_28_O_8_N2S	469.16475	25.4
	C_25_H_33_O_10_S	525.17576	32.36
	C_35_H_44_O_12_N_2_S	702.25905	31.72
	C_37_H_47_O_13_N_2_S	759.27629	28.89
	C_37_H_47_O_13_N_2_S	759.27631	29.46
	C_37_H_47_O13N_2_S	759.27619	29.73

^a^ Sirius/CS Finger-ID top hit.

^b^ GNPS spectral database match.

### Whole-genome sequencing of dehydrocurvularin-producing strains

Illumina short-read sequence data were generated from genomic DNA extracted from the three dehydrocurvularin producing *Alternaria* strains (DET2035, KAS5321, and KAS5743). An average of 28.2 million reads were generated per sample and whole-genome assemblies were produced. NCBI accession numbers for the newly generated assemblies are as follows: DET2035 = JAERPI000000000; KAS5321 = JAERNY000000000; KAS5743 = JAERND000000000. The resulting assemblies had an average sequence coverage of 125X and quality statistics were similar to previous *Alternaria* assemblies (Dettman & Eggertson 2021). The genome sizes ranged from 33.0 to 34.1 Mb, with a mean N50 of 897.8 Kb, and BUSCO analyses indicated the assemblies were over 99% complete (for whole-genome assembly statistics refer to Supplementary Section 7).

### Dehydrocurvularin BGC prediction and comparison to characterized BGCs

**FIGURE 3:**
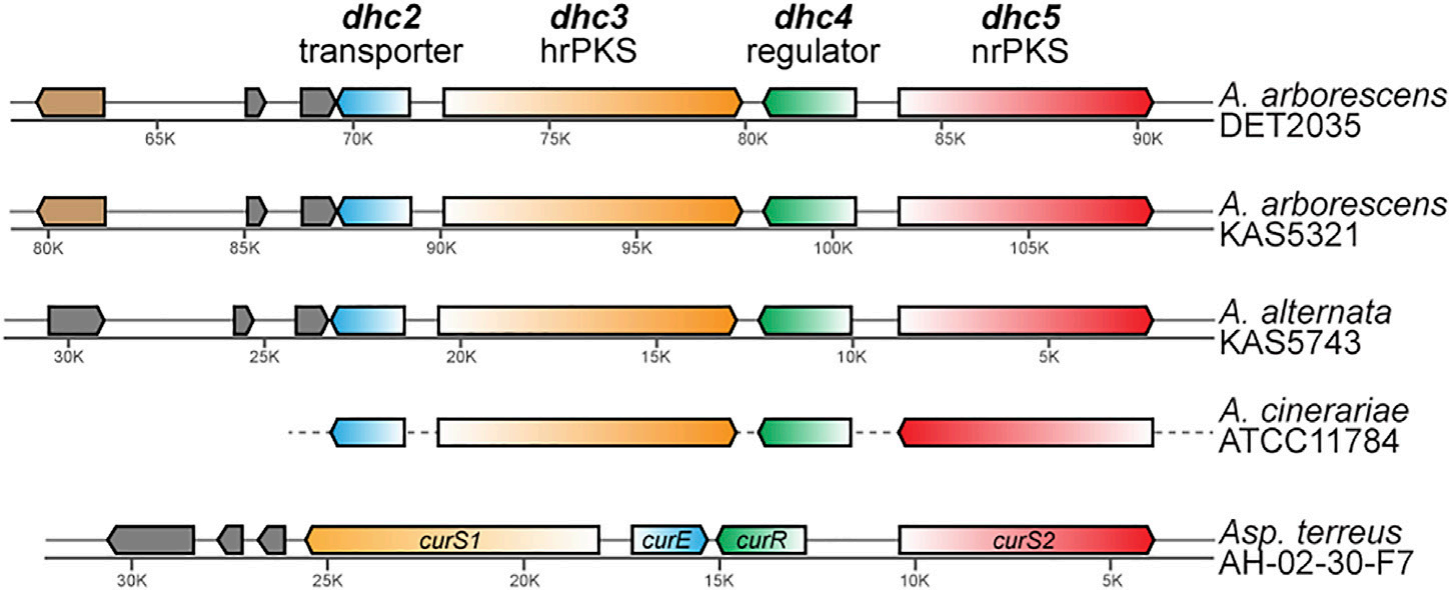
Comparison of homologous gene arrangement in the dehydrocurvularin BGC from the newly characterized *Alternaria* strains and previously characterized taxa (*A. cinerariae* and *Asp. terreus*). Genes with homologous sequence and predicted protein function are shown in the same color. The gene (light brown) that is unique to *A. arborescens* encodes for a glutamine-dependent NAD (+) synthetase. Genes shown in grey are not necessarily homologous and encode for hypothetical proteins that currently lack any functional predictions. Gene arrangements in *A. cinerariae* are as inferred by Cochrane et al. (2015).

Analysis of *ab initio* gene prediction, annotation, and secondary metabolite gene cluster identification with antiSMASH revealed that each of the three genomes had seven to eight type I polyketide synthase (PKS) containing BGCs. Only a single PKS BGC in each genome contained a highly reducing PKS—non reducing PKS pair (that is fairly rare in nature), and based on similarity to known dehydrocurvularin BGCs for *Aspergillus terreus* and *Alternaria cinerariae* (Xu et al., 2013; Cochrane et al., 2015), each showed evidence for involvement in dehydrocurvularin production. The predicted dehydrocurvularin BGCs ranged in size from 40.6 to 41.5 Kb in length and included between nine and ten genes. Only six of these genes were shared consistently by all three of the dehydrocurvularin BGCs (Figure 3).

Sequence comparison between the dehydrocurvularin BGC genes revealed clear homologous relationships for the four core genes identified here (Table 3). Functional annotation of conserved domains provided putative roles for four of the shared genes, all of which had plausible functions in dehydrocurvularin biosynthesis: 1) an efflux transporter in the major facilitator superfamily (MFS); 2) a highly reducing type I polyketide synthase (hrPKS); 3) a transcription factor with a Zn2-C6 fungal-type DNA-binding domain (regulator); and 4) a non-reducing type I polyketide synthase (nrPKS). Sequences of the new *Alternaria* dehydrocurvularin BGC genes were 94.4–95.5% similar to the *A. cinerariae* sequences. For consistency, we adopted the gene naming convention applied to *A. cinerariae* (*dhc2*, *dhc3*, *dhc4*, and *dhc5*) for the newly characterized biosynthetic genes in *A. arborescens* and *A. alternata*.

**TABLE 3:** Description and sequence homology comparison of four core genes involved in dehydrocurvularin (DHC) biosynthesis as observed from *A. arboresecens* and *A. alternata* strains and compared with published sequences from *A. cinerariae* and *Asp. terreus*.

	*A. arborescens & A. alternata*			*A. cinerariae* ^a^		*Aspergillus terreus* ^b^	
Gene product	Proposed gene name	Gene length (bp)	Protein length (aa)	Homolog	% Similarity (to DET2035)	Homolog	% Similarity (to DET2035)
Efflux transporter in the major facilitator superfamily (MFS)	*dhc2*	1868	581	*dhc2* (KT271471.1)	95.5%	*curE*	72.1%
Highly reducing type I polyketide synthase (hrPKS)	*dhc3*	7597	2389	*dhc3* (KT271472.1)	95.4%	*curS1*	72.4%
Transcription factor with a Zn2-C6 fungal-type DNA-binding domain (regulator)	*dhc4*	2353	764	*dhc4* (KT271473.1)	94.8%	*curR*	68.3%
Non-reducing type I polyketide synthase (nrPKS)	*dhc5*	6472	2080	dhc5 (KT271474.1)	94.4%	*curS2*	72.8%

^a^ Only coding sequences are available for *A. cinerariae*, so % similarity is calculated for coding regions only, not full length genes.

^b^ Sequence of the entire *Asp. terreus* DHC BGC is available on GenBank under one entry (JX971534.1). See Supplementary Material for coordinates of individual genes and other details.

The physical arrangement of the four core *dhc* genes within the BGC were quite similar between *Alternaria* spp. and the well-characterized *Asp. terreus*, with the main difference being the placement and transcriptional direction of *dhc2* (Figure 3). Although the full length sequence of the *A. cinerariae* dehydrocurvularin BGC is not available, the gene arrangements inferred by Cochrane et al. (2015) are shown for comparison. When considering the entire BGC, the gene content of the two *A. arborescens* strains were more similar to each other than to that of the *A. alternata* strain.

### Dehydrocurvularin BGC in other fungi

To explore the distribution of the dehydrocurvularin BGC in other fungal taxa, we searched the full NCBI nt database using each of the four core *A. arborescens* DET2035 *dhc* gene sequences as queries. BLASTn hits with greater than 80% sequence similarity were returned for only four taxa, all within the order Pleosporales: *A. atra*, *A. cinerariae*, *A. solani*, and *Pyrenophora tritici-repentis* (see Supplementary Section 8). Compared to BLASTn hits within Pleosporales (mean sequence similarity = 94.0%), the next closest matched sequences outside of Pleosporales had a much lower sequence similarity (mean = 74.8%) and were always from a species of *Aspergillus*. BLASTn searches using the known *A. cinerariae dhc* coding sequences returned similar results. For these reasons, we focused our attention on the dehydrocurvularin BGC in Pleosporales.

To expand the scope of our search, we gathered a panel of whole-genome assemblies of 281 Pleosporalean fungi from various public sources (for genome list see Supplementary Section 9). We maximized the phylogenetic diversity of taxa that were available at the time, with a total of 28 families, 65 genera, and 125 species being represented (including our three new *Alternaria* genomes). A local, custom database was created and BLASTn searches with *A. arborescens* DET2035 *dhc* gene sequences as queries revealed that only 25 of the 284 genomes (8.8%) contained the dehydrocurvularin BGC. Despite the wide diversity of taxa in our Pleosporalean genome database, the distribution of the dehydrocurvularin BGC was restricted to just one family, Pleosporaceae, and three genera within it: *Alternaria*, *Pyrenophora*, and *Stemphylium* (Table 4).

**TABLE 4:** BLASTn screening results for the dehydrocurvularin (DHC) BGC for the Pleosporaceae. Species with homologous BGCs are in bold text.

Species	# of genomes screened	# of genomes with DHC BGC detected	Species	# of genomes screened	# of genomes with DHC BGC detected
*Alternaria alstroemeriae*	1	0	*Bipolaris cookei*	1	0
***Alternaria alternata***	**37**	**1**	*Bipolaris maydis*	5	0
***Alternaria arborescens***	**11**	**2**	*Bipolaris oryzae*	2	0
***Alternaria atra***	**2**	**2** ^a^	*Bipolaris sorokiniana*	8	0
*Alternaria brassicae*	1	0	*Bipolaris victoriae*	1	0
*Alternaria brassicicola*	3	0	*Bipolaris zeicola*	2	0
*Alternaria burnsii*	1	0	*Curvularia eragrostidis*	1	0
*Alternaria capsici*	1	0	*Curvularia geniculata*	2	0
***Alternaria carthami***	**1**	**1**	*Curvularia lunata*	3	0
***Alternaria consortialis***	**1**	**1**	*Curvularia papendorfii*	1	0
*Alternaria crassa*	1	0	*Curvularia sp.*	1	0
*Alternaria dauci*	1	0	*Decorospora gaudefroyi*	2	0
*Alternaria gaisen*	3	0	*Exserohilum rostratum*	1	0
*Alternaria gansuensis*	1	0	*Exserohilum turcicum*	2	0
*Alternaria longipes*	2	0	*Neocamarosporium betae*	1	0
***Alternaria macrospora***	**1**	**1**	*Paradendryphiella salina*	1	0
*Alternaria panax*	1	0	*Pyrenophora graminea*	1	0
***Alternaria porri***	**1**	**1** ^a^	*Pyrenophora seminiperda*	1	0
*Alternaria rosae*	1	0	*Pyrenophora teres f. maculata*	6	0
***Alternaria solani***	**3**	**3**	*Pyrenophora teres f. teres*	12	0
*Alternaria sp.*	1	0	***Pyrenophora tritici-repentis***	**11**	**11**
*Alternaria tangelonis*	1	0	*Stemphylium lycopersici*	3	0
***Alternaria tomatophila***	**1**	**1** **^a^**	***Stemphylium vesicarium***	**3**	**1**

^a^ For one genome, the full length DHC BGC was not on a single contig. Individual genes, or separate genes in the cluster, may have been split across multiple contigs.

The dehydrocurvularin BGC was detected in only nine of the 23 screened species of *Alternaria* (*A. alternata*, *A. arborescens*, *A. atra*, *A. carthami*, *A. consortialis*, *A. macrospora*, *A. porri*, *A. solani*, and *A. tomatophila*), totalling ten species when the known BGC from *A. cinerariae* is included. In three dehydrocurvularin-positive *Alternaria* genomes, the BGC was split across multiple contigs (see Supplementary Section 10). In these cases, the cluster was typically close to the edge of a fairly short contig, suggesting that BGC contig splitting was caused by incomplete assemblies rather than the genes being located in non-adjacent regions of the genome. Our largest sample sizes were for *A. alternata* (*n* = 37) and *A. arborescens* (*n* = 11), however, all of the previously published genomes for *A. alternata* and *A. arborescens* lacked the dehydrocurvularin BGC. The only strains from these species that possessed the dehydrocurvularin BGC were the three strains that were denoted from our untargeted metabolomics screening (that were newly characterized and sequenced here). Our metabolic screening of strains, therefore, was essential for identifying the extremely rare curvularin producers in *A. alternata* and *A. arborescens*.

In contrast to the genus *Alternaria*, the pattern of dehydrocurvularin BGC distribution in the genus *Pyrenophora* was very consistent within the genus. The dehydrocurvularin BGC was found in only one of the screened species, *Pyrenophora tritici-repentis*, but it was detected in 100% of the 11 available genomes of that species. In *Stemphylium*, the dehydrocurvularin BGC was found in only one out of the six available genomes for the genus (*S. vesicarium*); only two species, *S. lycopersici* (*n* = 3) and *S. vesicarium* (*n* = 3) were represented in the database.

### Genomic location of dehydrocurvularin BGC in *Alternaria*

**FIGURE 4:**
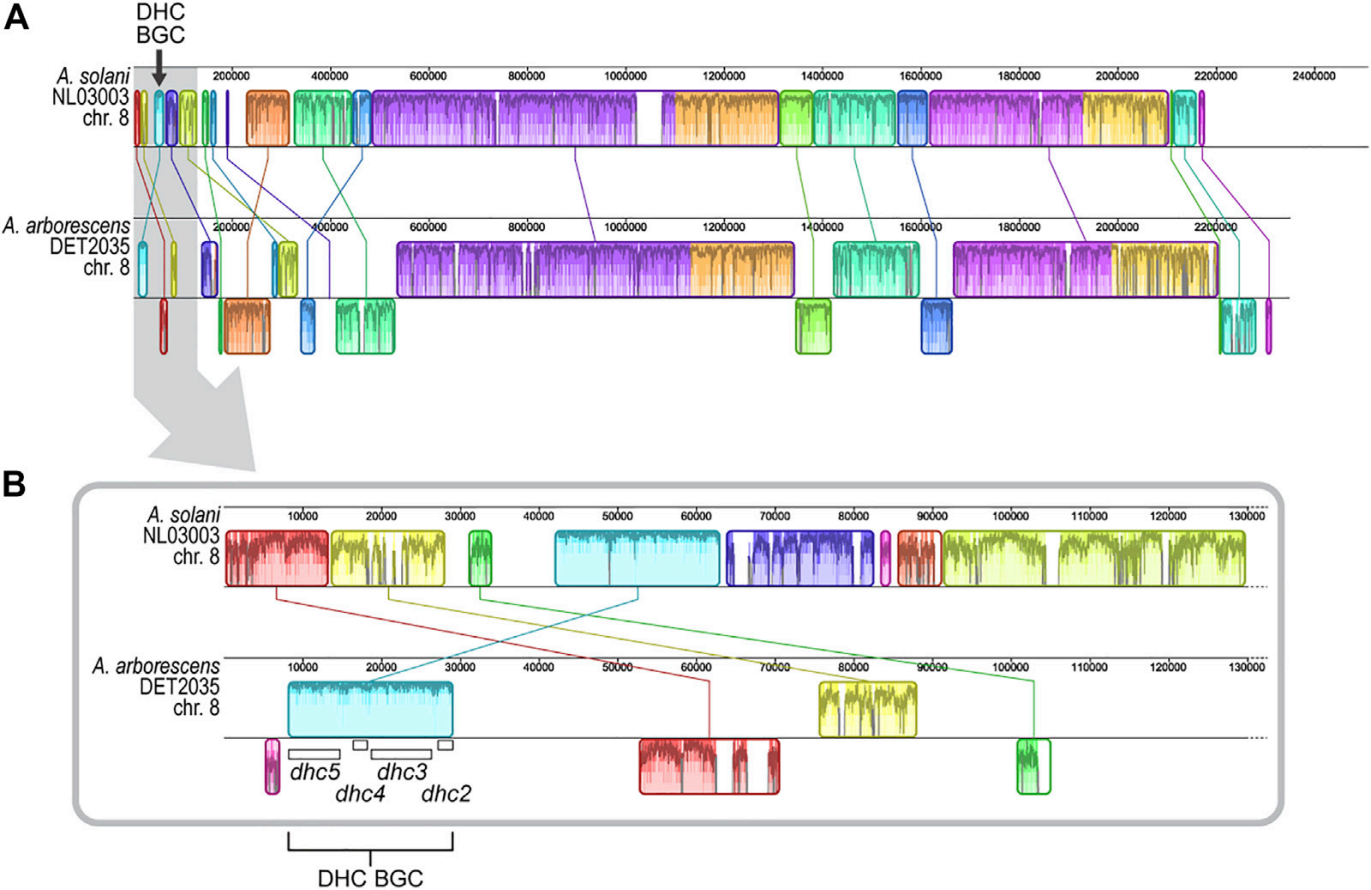
Alignment of genome regions from *A. arborescens* DET2035 and *A. solani* NL03003. Locally collinear blocks (LCBs) are shown in matching colors and linked by lines. LCBs shown below the horizontal centre line in *A. arborescens* DET2035 have inverse orientation in comparison to *A. solani* NL03003. Regions outside LCBs lack detectable homology between the genomes. Within each LCB, the height of the similarity plot corresponds to the average level of sequence conservation in that region, and sections in white are unique to that specific genome. **(A)** Alignment of full-length chromosome 8. **(B)** Alignment of the first 130 Kb of chromosome 8 (area highlighted in grey in panel A). The location of the four *dhc* genes are shown.

The chromosomal genomic location of previously characterized dehydrocurvularin BGCs in *A. cinerariae* and *Asp. terreus* cannot be determined because chromosomal length genome sequences were not available for the studied strains (Xu et al., 2013; Cochrane et al., 2015). When comparing the positional coordinates of the dehydrocurvularin BGCs in positive genomes of the *A. alternata* and *A. arborescens* strains studied here, we found that the BGC was typically near a contig edge. The dehydrocurvularin BGC boundary was, on average, within the first or last 10.0% (SE 1.5%) of the contig sequence. Restricting the analysis to genomes with chromosome-level assemblies from long-read data revealed the BGC is located in subtelomeric regions of the chromosome. In the only available *Alternaria* chromosome-level assembly (*A. solani* NL03003), the dehydrocurvularin BGC is located in the first 1.7% of the 2.51 Mb chromosome 8 (Figure 4).

We generated 22.43 Gb of long-read sequence data (Oxford Nanopore) for *A. arborescens* DET2035 and produced a telomere-to-telomere assembly of chromosome 8 (NCBI accession number CP104802). Similar to *A. solani*, the dehydrocurvularin BGC in *A. arborescens* DET2035 is located 8.3 kb from the chromosome 8 telomeric edge. Full chromosome alignment of *A. solani* NL03003 and *A. arborescens* DET2035 (Figure 4) showed that the chromosomal structure was fairly well conserved, with large locally colinear blocks of sequence in the central regions. As expected, the subtelomeric regions appear more variable, with smaller conserved blocks and evidence of rearrangements, inversions, and non-homologous regions.

Next, we examined the genomic regions adjacent to the dehydrocurvularin BGC for evidence of transposable elements. Interestingly, fragments of a Gypsy transposon were immediately adjacent to the dehydrocurvularin BGC (telomere-proximal), with one of the long terminal repeats occurring only 318 nucleotides from the end of the *dhc5* gene. On the other side of the dehydrocurvularin BGC (telomere-distal), fragments of a hAT transposon and a DDE transposase were found within 7.8–15.4 Kb of the *dhc2* gene.

## Discussion

To date, dehydrocurvularins have been isolated from a number of phytopathogenic fungal species, namely *Alternaria, Aspergillus, Cochliobolus/Curvularia, Nectria,* and *Penicillium* spp. (Musgrave, 1956; Hyeon et al., 1976; Robeson et al., 1985; Ghisalberti & Rowland, 1993; He et al., 2004; Zhan et al., 2004; Gutiérrez et al., 2005) and the geographical distribution of these instances spans most of the world (with production observed in isolates from Chile, Japan, Europe, Egypt, North America, and Korea) (Musgrave, 1956; Munro et al., 1967; Kusano et al., 2003; Gutiérrez et al., 2005; Aly et al., 2010; Jeon et al., 2010; Bashyal et al., 2017). *Alternaria* spp*.* that are reported to produce dehydrocurvularins include *A. cinerariae, A. zinniae*, *A. macrospora, A. tomatophila*, and an *Alternaria sp.* isolate from the leaves of *Acaia mangium* (Hyeon et al., 1976; Robeson & Strobel, 1981; Robeson et al., 1985; Vurro et al., 1998; Jeon et al., 2010). Additionally, two chlorinated dehydrocurvularin analogs were previously isolated from an *Alternaria* sp*.* isolated from the leaf tissue of *Astragalus lentinginosus* (Bashyal et al., 2017). The findings of the present study represent the first isolation of dehydrocurvularin from verified *A. alternata* or *A. arborescens* isolates. Additionally, this represents the first report of sumalarins A and C in any *Alternaria* species.

Dehydrocurvularin exhibits broad, non-specific phytotoxicity against rice (*Oryza sativa*), lettuce (*Lactuca sativa*), millet (*Panicum miliaceum*), common Zinnia (*Zinnia elegans*)*,* Canadian thistle (*Cirsium arvense*), crabgrass (*Digitaria sanguinalis*)*,* Noogoora burr (*Xanthium occidentale*) and a number of other agronomic weedy plant species (Robeson & Strobel, 1981; Vurro et al., 1998; Kusano et al., 2003; Gutiérrez et al., 2005; Jiang et al., 2008). Mechanistic phytotoxicity assays are lacking beyond one study which linked dehydrocurvularin exposure to disruption of mitosis in garlic root tip cells (Jiang et al., 2008). However, symptoms of dehydrocurvularin exposure have been linked to reductions in seed germination, root growth, epicotyl growth, cotyledon growth, and the appearance of necrotic lesions and/or chlorosis in leaf tissue assays (see above citations). Among the dehydrocurvularin analogs tested, dehydrocurvularin is consistently associated with the most pronounced phytotoxic response. In addition to phytotoxicity, dehydrocurvularin exhibits antifungal activity through suppression of sporulation and spore germination (Hyeon et al., 1976), modest antibacterial activity against several Gram-positive and Gram-negative bacteria (such as *Bacillus subtilis, Staphylococcus aureus,* and *Escherichia coli*; Caputo and Viola, 1977), and significant nematicidal activity against *Meloidogyne graminicola* (Xiang et al., 2020). Dehydrocurvularin also disrupted mitosis in sea urchin embryos (Kobayashi et al., 1988). Given the broad toxicity profile of this molecule, it is reasonable to conclude that dehydrocurvularin is not a host-specific toxin and will likely have a toxic effect upon the producing organism when produced at high enough levels (such as during *in vitro* cultivation). It is currently unknown if the dehydrocurvularins will impart increased disease virulence of the producing organism or whether production will provide a fitness advantage in some other context.

The untargeted metabolomics profiling experiments showed various dehydrocurvularin-associated molecules were produced in high abundance by our *A. alternata* and *A. arborescens* strains, including dehydrocurvularin, 11-hydroxycurvularin, 11-methoxycurvularin and sumalarin C. While curvularin was detected, it was not as a major product of DET2035. Various low intensity dehydrocurvularin-associated mass features were also detected, many of which were predicted to contain a sulfur atom and were annotated as cyclothiocurvularins, sulfoxicurvularins and nitrogen-containing curvularins. This large family of dehydrocurvularin-associated molecules likely arises from spontaneous addition of various nucleophiles to the enone group of dehydrocurvularin followed in some cases by further chemistry.

11-hydroxycurvularin and 11-methoxycurvularin are presumably formed by spontaneous addition of water or methanol to dehydrocurvularin, likely during handling of the fungal extracts as has been previously proposed (He et al., 2004). This spontaneous Michael addition is supported by the observation of duplicate sets of isomeric mass features at different retention times for each due to the non-stereo controlled formation of the α- and β-stereoisomers. Similarly, the sumalarins, originally isolated from *Penicillium sumatrense* MA-92 are proposed to arise from Michael addition of the cysteine metabolite 3-mercaptolactate, and may play a role in detoxification of dehydrocurvularin by the producing fungus during *in vitro* cultivation (Meng et al., 2013). The related cyclothiocurvularins have been isolated from *Penicillium* sp. isolate DRF2 (de Castro et al., 2016). The cyclothiocurvularins have unambiguously been shown to form from *in vitro* treatment of dehydrocurvularin with cysteine derived mercaptopyruvate. de Castro et al. (2016) also observed formation of the α- and β-stereoisomers from attack on the enone *in vitro*, which is consistent with our data showing duplicate isomeric mass features for cyclothiocurvularins. These data and prior precedent are consistent with spontaneous non-enzyme catalyzed Michael addition generating these metabolites.

Thiol-dehydrocurvularin Michael adducts can undergo additional processing. For example these thioethers are known to undergo oxidation to generate sulfoxides. Cyclothiocurvularin is readily oxidized to the sulfoxide cyclosulfoxicurvularin, which has been isolated along with cyclothiocurvularin (de Castro et al., 2016). Similarly we observed a mass feature consistent with this sulfoxide (Table 2). In addition we detected mass features consistent with oxidized sumalarin C (sulfoxicurvularin, Table 2). The possibility that sulfoxidized analogs are produced during sample handling and analysis cannot be ruled out at this time, and we note that these forms were detected only at low intensities in the fungal extracts.

We detect a number of mass features that were annotated as cysteinylglycine-, cysteine-, and N-acetylcysteine- Michael adducts of dehydrocurvularin. The detection of these nitrogen-containing dehydrocurvularins has not been described to date. We propose that these are a by-product of glutathione addition to dehydrocurvularin. Although we did not detect any *m/z* matching dehydrocurvularin-S-gluthathione in this experiment, cysteine, cysteinylglycine, and N-acetylcysteine conjugates are known to be generated from the glutathione adducts in fungi including *Sclerotinia sclerotiorum* (Chen et al., 2020).

Conjugation with thiol nucleophiles likely represents a detoxification pathway for dehydrocurvularin. Electrophilic compounds, like dehydrocurvularin, can be detoxified *via* the mercapturate conjugation pathway. For example, deoxynivalenol, an electrophilic *Fusarium graminearum* mycotoxin, is converted into deoxynivalenol-S-glutathione, deoxynivalenol-S-cysteine and deoxynivalenol-S-cysteinylglycine for detoxification by wheat tissue (Kluger et al., 2013). Similarly, the Brassicales plants defensive compounds containing electrophilic isothiocyanates are detoxified by pathogenic *S. sclerotium* isolates *via* the mercapturate conjugation pathway, resulting in detection of -cysteine, -cysteinylglycine, and -N-acetylcysteine conjugates (Chen et al., 2020). Our data thus suggests that a mercapturate conjugation detoxification pathway is also activated in the case of dehydrocurvularin-producing *Alternaria* strains.

Conjugation of dehydrocurvularin with mercaptolactate or mercaptopyruvate to form sumalarin C or cyclothiocurvularins, respectively, could represent an additional detoxification route. This has been proposed for cyclothiocurvularins because both cyclothiocurvularin and sulfoxicyclothiocurvularin have significantly reduced toxicity towards cancer cell lines in comparison to dehydrocurvularin (de Castro et al., 2016). Interestingly, sumalarin C was found to be comparably toxic towards cancer cell lines relative to dehydrocurvularin (Meng et al., 2013). An alternative explanation for the reported cytotoxicity of sumalarin C could be that the Michael addition of mercaptolactate to dehydrocurvularin is reversible in biological contexts, as has been found for the aculeatusquinone C conjugate of dehydrocurvularin (Shang et al., 2016). Similarly, 11-methoxycurvularin has been reported to be highly unstable and easily dehydrated to give dehydrocurvularin during separation on preparative TLC plates and on HPLC columns (Lai et al., 1991). The presence of numerous, potentially reversible conjugates of dehydrocurvularin raises the intriguing possibility that conjugated forms of dehydrocurvularin could act as “masked” or modified toxins which are metabolized or spontaneously revert to toxic dehydrocurvularin forms in host cells. This hypothesis must be tempered however by the observation that thiol-adducts are typically formed irreversibly by Michael addition to enones under biological relevant conditions (Jackson et al., 2017). Regardless, the multitude of proposed “detoxification” products detected in this experiment and previously reported in the literature (de Castro et al., 2016) raises the question of self-toxicity for dehydrocurvularin-producing isolates, and offers a potential rationale for selective pressures favoring loss of the dehydrocurvularin cluster in certain contexts.

The sporadic distribution of the dehydrocurvularin BGC across a wide diversity of taxa demonstrated how rare dehydrocurvularin production may be in the order Pleosporales. We discovered the dehydrocurvularin BGC in only one of 28 families (3.6%), and in only three of 65 genera (4.6%; *Alternaria*, *Pyrenophora*, and *Stemphylium*). Within genera, the prevalence of the dehydrocurvularin BGC was slightly higher; one of five *Pyrenophora* species (20%), and ten of the 24 *Alternaria* species (41.7%), had evidence of the dehydrocurvularin BGC in their genomes. Although this is the most comprehensive survey possible at the time, we fully acknowledge that discovery is limited to the breadth of the database, and that some dehydrocurvularin-positive taxa are likely to be missing. More intensive sampling at all taxonomic levels is required before we can propose predictive guidelines for which taxa may possess the dehydrocurvularin BGC. Interestingly, the dehydrocurvularin BGC was not detected in any of the eight examined genomes from the genus *Curvularia*, the namesake of curvularin.

The order and orientation of the four core genes within the dehydrocurvularin BGC were well conserved within the *Alternaria*, *Pyrenophora*, and *Stemphylium* genomes. The location of the dehydrocurvularin BGC in the greater genomic context was much more difficult to determine with certainty. Based on the positional coordinates of complete BGCs, the cluster was typically located near the terminal ends of a contig, and in some cases, the contig was so short that the dehydrocurvularin BGC comprised nearly the entire contig. This pattern suggests the dehydrocurvularin BGC is flanked by genomic regions that are difficult to assemble with short-read sequence data, such as regions with repetitive elements such as transposable elements or telomeres. To overcome this problem, we generated long-read sequence data and created a telomere-to-telomere assembly of the dehydrocurvularin BGC-containing chromosome from *A. arborescens* DET2035. Comparative analyses with the only other dehydrocurvularin-positive *Alternaria* strain with a chromosome-level assembly (*A. solani* NL03003) confirmed the dehydrocurvularin BGC was located in a subtelomeric region of chromosome 8 in both strains (Figure 4).

Subtelomeric regions of fungal chromosomes are commonly repeat-rich and gene-poor, with elevated rates of recombination and mutation, resulting in overall elevated rates of genomic evolution at chromosome ends (Brown et al., 2010; Rahnama et al., 2020). As a consequence, the high rate of gene content gain/loss within lineages can lead to high levels of gene content diversity and divergence between lineages in subtelomeric regions, thereby providing the raw material for adaptive evolution. Our preliminary comparative analysis was consistent with this prediction, in that subtelomeric regions did have smaller conserved blocks and more rearrangements, inversions, and non-homologous regions. We note that our sample size is only two chromosomes, and that additional full chromosome assemblies are needed to properly assess relative rates of subtelomeric variation in *Alternaria*.

Due to the sporadic occurrence of the dehydrocurvularin BGC as observed within *A. arborescens* and *A. alternata*, questions arise with regards to the origin of the dehydrocurvularin BGC within the genus. For example, is the pattern of the dehydrocurvularin BGC distribution in Pleosporalean fungi more consistent with horizontal gene transfer or vertical gain/loss during lineage evolution? In cases like *Pyrenophora tritici-repentis*, where all examined genomes have the dehydrocurvularin BGC, the most logical explanation is a single gain-of-function event that occurred shortly after the species diverged from the other *Pyrenophora* species. In contrast, the disjunct distribution observed in *Alternaria* and *Stemphylium* suggests both options are possible. These three genera are known to commonly infect the same agricultural hosts (e.g. wheat), creating opportunity for close physical contact. An intriguing observation made from the telomeric synteny comparison in this study (Figure 4) was that the *dhc* four-gene collinearity block appears to exhibit consistently less divergence compared to all other nearby collinear blocks. This observation contradicts our understanding of the high rate of mutation associated with subtelomeric regions, and may suggest either a recent horizontal transfer of the dehydrocurvularin BGC has occurred, or that this BGC region is under strong negative selective pressure. However, the mechanism by which genetic information would be transferred between genera is not clear, so at this point, vertical gain/loss during lineage evolution is the preferred hypothesis. More examples of dehydrocurvularin BGC-positive *Alternaria* and *Stemphylium* strains need to be discovered and chromosomal-level genome assemblies acquired, to facilitate more detailed analyses of the phylogenetic relationships among strains and of the chromosomal organization of the BGC themselves. Only then will the evolutionary history of these dehydrocurvularin BGCs be revealed.

Extensive whole genome-based screening for unique secondary metabolite biosynthetic gene clusters within species is, at the moment, cost prohibitive; although costs for whole-genome sequencing are on the decline. Untargeted metabolomics screening offers a more affordable option to screen larger numbers of strains. As a proof of concept for the genus *Alternaria*, the utility of untargeted metabolomics profiling has proven to be successful at detecting unique metabolite production within an intra-specific population—metabolite production that can be linked to biosynthetic gene clusters from informed selection-based whole-genome screening. A caveat of untargeted metabolomics screens of fungal populations is that not all secondary metabolite biosynthetic gene clusters are expressed under a single given *in vitro* cultivation condition. Utilizing multiple media conditions is therefore necessary to stimulate the maximum breadth of secondary metabolite diversity from the screening population as minor variations in environmental and/or nutritional conditions have the potential to impact the diversity of secondary metabolism in fungi (Bode et al., 2002; Bills et al., 2008). However, increasing the number of culture conditions quickly becomes a limiting factor, especially when sufficient replication is required for statistical analyses. In the case of this current study, an initial pilot study was first performed using eight different media and a subset of the strains (data not shown)—in the end four growth media were selected as they covered the breadth of secondary metabolite production as observed from the initial eight.

The untargeted metabolomics screening results generated in this study can be utilized for further informed strain selection for targeted whole-genome sequencing, in particular to investigate production trends for other *Alternaria* secondary metabolites. Interesting patterns of production frequency variance were observed between strains and between the secondary metabolites themselves; some were detected in most, but not all of the 36 strains profiled (i.e. altersetin, alternariol, alternariol monomethyl ether, and tenuazonic acid) while others were limited to subsets of the population and production frequency was limited to only a few media (i.e. tentoxin, dihydrotentoxin, altenuene and altenusin). This observed variability in secondary metabolite production is consistent with previous targeted secondary metabolite screening of *A. alternata* and *A. arborescens* populations (Patriarca et al., 2019). However from our screening results, it is unclear if the non-producing strains lack the associated BGC in the genome; or if the BGC resides in a subtelomeric region of the chromosome and due to transposable element insertion has been pseudogenized and rendered non-functional; or if the growth conditions provided were insufficient to trigger expression of the BGC. Metabolomics guided strain selection for whole-genome sequencing (both short read and long read), chromosomal level assembly, and BGC comparison between producing and non-producing strains will provide the necessary insight to answer these questions. Understanding the underlying mechanism to account for the variability in production of alternariol, alternariol methyl ether, tenuazonic acid, and tentoxin, is of particular interest as these mycotoxins contaminate agricultural products and are of concern for regulatory and advisory agencies such as the European Food Safety Authority (EFSA, 2011; Solfizzo, 2017). The biosynthetic gene clusters for each of these mycotoxins of concern are currently known (Saha et al., 2012; Li et al., 2016; Sun et al., 2022). Additional research efforts focused towards BGC comparison as outlined above are currently underway, along with an expanded strain screening of *Alternaria* section *Alternaria* to explore the occurrence of unique secondary metabolite production.

## Data Availability

The datasets presented in this study can be found in online repositories. The names of the repository/repositories and accession number(s) can be found in the article/Supplementary Material.
