# Supplementary material for: Untargeted metabolomics screening reveals unique secondary metabolite production from Alternaria section Alternaria
Source: Front Mol Biosci. 2022 Nov 24;9:1038299. doi: 10.3389/fmolb.2022.1038299 (PMC9731300; doi:10.3389/fmolb.2022.1038299)
Supplement: Supplementary file 1 [file DataSheet1.PDF]

Supplementary Information

1 Media Formulations

1.1 Composition of the CYS80 medium (1 L volume)

Sucrose	80.0g
Corn meal	50.0g
Yeast extract	1.0g
$MgSO_4 \cdot 7H_2O$	0.5g
Agar	20.0g
Nanopure water	to 1.0L

1.2 Composition of the DRYES medium (1 L volume)

Sucrose	150.0g
Yeast extract	20.0g
$MgSO_4 \cdot 7H_2O$	0.5g
Dichloran (0.2% in EtOH)	1.0mL
Rose bengal (5%, w/v)	0.5mL
Agar	20.0g
Nanopure water	to 1.0L

1.3 Composition of the MMK2 medium (1 L volume)

Mannitol	40.0g
Yeast extract	5.0g
Murashige and Skoog Salts	4.3g
Agar	20.0g
Nanopure water	to 1.0L

1.4 Composition of the ZM2 medium (1L volume)

Molasses	5.0g
Oatmeal	5.0g
Sucrose	4.0g
D-glucose	1.5g
$CaCO_3$	1.5mg
Edamin	0.5g
$(NH_4)_2SO_4$	0.5g
Agar	20.0g
Nanopure water	to 1.0L

1.5 Composition of the PDA medium (1 L volume)

Potato extract	4.0g
Dextrose	20.0g
Agar	15.0g
Nanopure water	to 1.0L

2 Chemical and Solvent List

**Chemical**	**Manufacturer**
Ethanol (EtOH)	Thermo-Fisher Scientific
Ethyl acetate (HPLC-grade)	EMD Millipore
Methanol (LCMS-grade)	VWR Chemicals
Acetonitrile (Optima, LCMS-grade)	Thermo-Fisher Scientific
Water (Optima, LCMS-grade)	Thermo-Fisher Scientific
Nanopure water	Thermo-Fisher Scientific, Barnstead Nanopure
Sucrose	Anachemia
Corn meal	Unico
Yeast extract	Oxoid
$MgSO_4 \times 7H_2O$	BDH
Agar	VWR
Dichloran	Sigma-Aldrich
Rose bengal	Sigma-Aldrich
Mannitol	Sigma-Aldrich
Murashige and Skoog Salts	Phytotechnology Laboratories
Molasses	Grandma's®, B&G Foods Inc.
Oatmeal	Quaker Oats Company
D-glucose	Sigma-Aldrich
$CaCO_3$	Thermo-Fisher Scientific
Edamin	Sigma-Aldrich
$(NH_4)_2SO_4$	Thermo-Fisher Scientific
Formic acid	Sigma-Aldrich

3 Phylogenetic placement of *Alternaria* strains used for metabolomics profiling.

Maximum likelihood tree constructed from combined sequence analysis from three loci: *ASA-10*, *ASA-19*, and *rpb2* (RNA polymerase II second largest subunit). The fifteen taxa highlighted in red are examples of taxa included in the metabolomics profiling. Red arrows indicate the placement of dehydrocurvularin-producing strains. Bootstrap support (percentages) are displayed on major branches. Substitution model selection and tree construction were performed with IQ-TREE (2.0). The MODELFINDER module was used to choose the best-fit substitution model and the optimal

partitioning scheme for the three datasets combined was applied (-m MFP+MERGE).

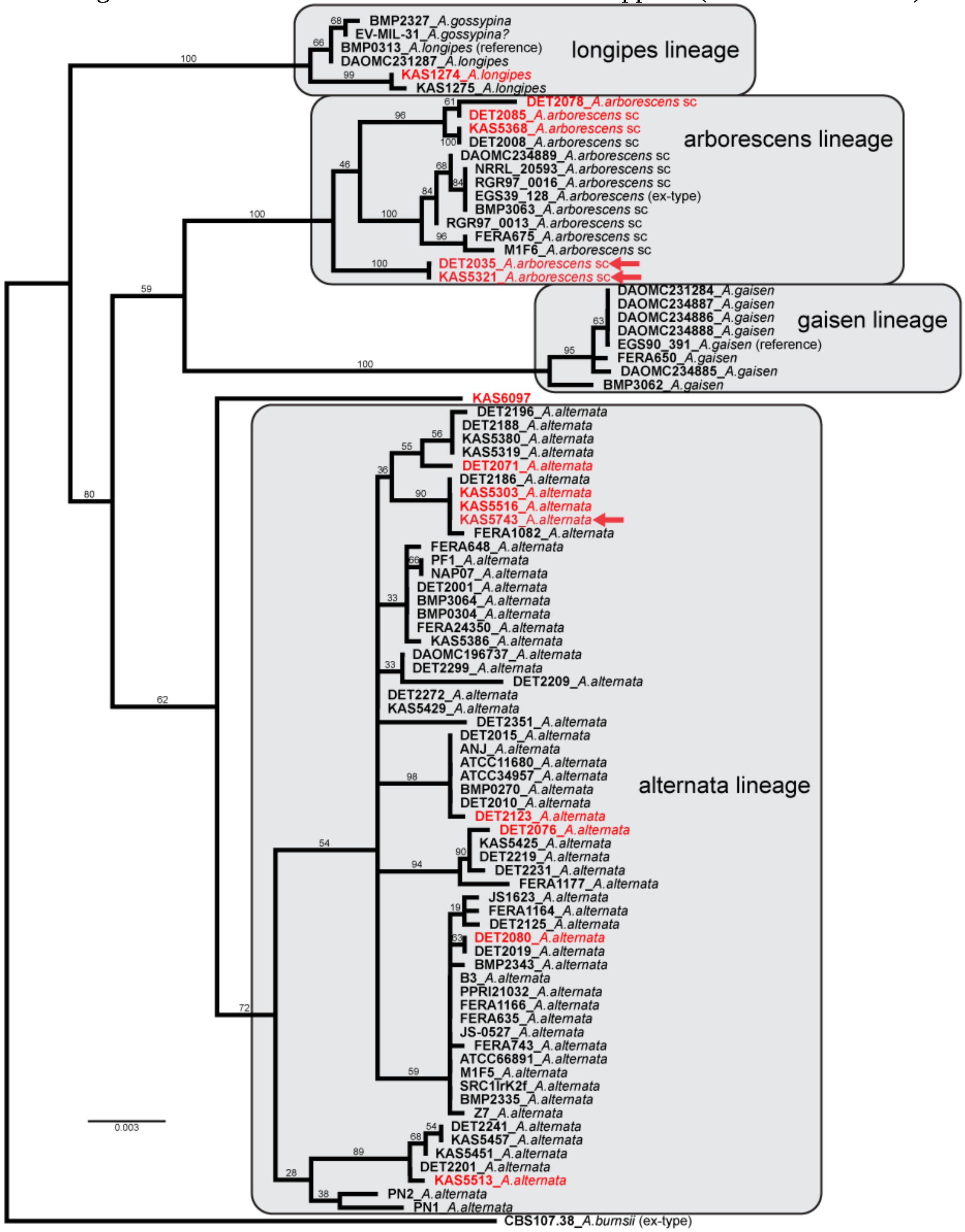

4 Dehydrocurvularin production and structure elucidation

4.1 Scaled up cultivation of DET2035

Alternaria arborescens strain DET2035 was 3-point inoculated onto 300 Petri plates of CYS80 agar and incubated at 25 °C with a light/dark cycle of 8/16 hrs for 14 days. Plates were subsequently

harvested by cutting the culture into squares (1 cm x 1 cm) using a scalpel into a 5 x 2 L Erlenmeyer flasks to a volume of 600 mL in each flask, to which 1300 mL of ethyl acetate (EtOAc) was then added and covered with aluminum foil. In a chemical fume hood, extractions were performed on a rotary shaker at 160 RPM for 4 hours. The resulting solvent extracts were vacuum filtered through filter paper (Whatman 5230-110, Grade 230, diam 100 mm) concentrated in vacuo using a rotary evaporator (IKA RV10).

4.2 Extraction and purification of dehydrocurvularin

A de-fatting step was performed by re-suspending the extract in methanol and carrying out a liquid:liquid partition with an equal volume of hexanes in a 2 L separatory funnel. The methanol fraction was retained and concentrated in vacuo. The dried bulk extract was re-suspended in MeOH and filtered through a 0.45 μm PFTE Acrodisc (Pall Corporation, MI, USA) syringe filter prior to fractionation by preparative HPLC (1260 Infinite Series, Agilent Technologies, CA, USA coupled to Fraction collector) on a Phenomenex Kinetex C18 100Å LC column (100 x 30.0 mm, 5 μm). Fractionation of the extract was carried out iteratively using various ACN (+0.05% formic acid) : H_2O (+0.05% formic acid) gradients to obtain DHC in a semi-purified state. Each fraction was evaporated in vacuo to dryness using a GeneVac EZ-Elite 2 (SP Scientific, Ipswich, UK). An aliquot of each fraction was retained for analytical UPLC-HRMS/DAD/CAD analysis. Final purification of DHC was performed by thin layer chromatography using silica gel plates (GF_{254}, Merck, 20 x 20 cm with 0.25 mm thickness) and 5% MeOH in chloroform as the mobile phase.

4.3 NMR conditions

^{1}H-NMR, ^{13}C-NMR, and HSQC data were obtained using a Bruker Avance HD 600 of the NMR Facility of the University of Ottawa. The ^{1}H-NMR data was obtained at 600 MHz and ^{13}C-NMR data was obtained at 150 MHz. To allow for direct comparison to literature, DHC was re-suspended in CD_3OD for NMR analyses.

4.4 Dehydrocurvularin molecular structure and Carbon numbering

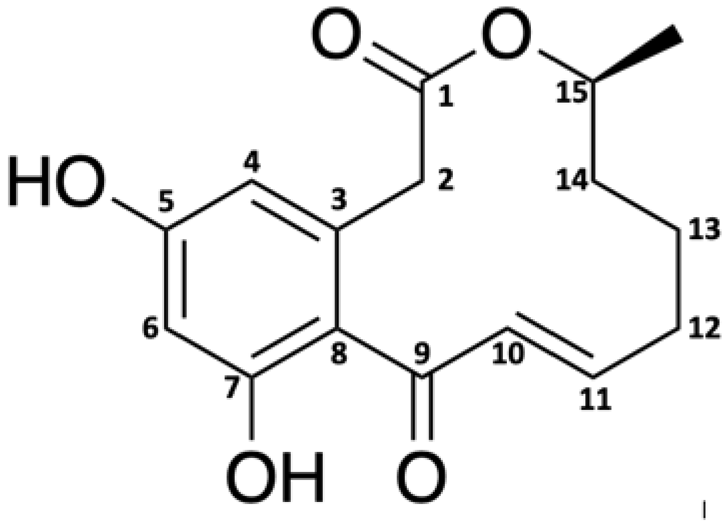

4.5 Dehydrocurvularin structure elucidation

To confirm the molecular structure of unknown 1 as dehydrocurvularin, ^{1}H-NMR, ^{13}C-NMR, and HSQC analysis were performed. The ^{1}H-NMR analysis of the spectra revealed two aromatic protons (δ_H of 6.30 ppm and δ_H of 6.30 ppm) as well as two alkene protons (δ_H of 6.51 ppm and δ_H of 6.59 ppm). The alkene protons show a large coupling constant (J=15.4 Hz), suggesting a *trans* olefin. Two olefinic protons form the AB portion. Protons at δ_H 3.74 ppm and 3.45 ppm each have identical coupling constants (J=16.6 Hz), consistent with geminal coupling. The three protons at δ_H of 1.20 ppm are a doublet with J=6.4 Hz, consistent with a methyl group coupled to a single adjacent hydrogen.

Inspection of the ^{13}C-NMR revealed 6 aromatic carbons (C3-C8). δ_C of 162.02 and 162.30 ppm are consistent with aromatic carbons experiencing the added de-shielding of being bound to an OH group. The resonance of 173.04 ppm (C1) is consistent with a carbonyl carbon that is experiencing de-shielding from at least one neighboring heteroatom (an ester group). Furthermore, the resonance at 72.4 (C15) is consistent with a saturated carbon bound to the oxygen. Combined, these two signals suggest an ester. The observed resonance at 200.08 ppm (C9) suggests there is a ketone in the structure. The resonances observed at 133.4 ppm and 154.4 ppm (C10 and C11, respectively) are consistent with an olefin connected to an electron withdrawing group. With the ketone, this suggests an α,β - unsaturated ketone is present. The remaining resonances of 34.2 (C12), 25.5 (C13), 35.2 (C14), and 20.4 (CH3) are all consistent with alkane chain carbons.

Analysis of the HSQC confirms that the carbons at δ_C of 200 ppm, 173 ppm, 137.3 ppm, 162.02, 162.30, and 117.88 ppm have no attached hydrogens. The carbons are 112.20 ppm and 102.8 ppm are attached to the aromatic protons at δ_H of 6.30 and 6.26 respectively. The alkene protons at δ_H of 6.50 ppm and 6.59 ppm are connected to the carbons at 133.4 and 154.4, respectively. Lastly, the carbon δ_C 74.3 ppm is indicative of a carbon with a single bond to an electronegative heteroatom and is connected to the proton at δ_H 4.82 ppm.

Four alkane carbons are clearly identifiable. The carbon at δ_C 42.4 ppm couples to the protons at δ_H 3.74 ppm and 3.46 ppm. The carbon at δ_C 35.2 ppm couples to the protons at δ_H 1.91 – 1.81 ppm and

1.64 – 1.55 ppm. The carbon at δ_C 34.2 ppm couples to the protons at δ_H 2.41 ppm and 2.36 – 2.26 ppm. Finally, the methyl carbon at δ_C 20.4 ppm couples to three protons at δ_H 1.20 ppm.

These data are consistent with the functional groups found in dehydrocurvularin, a tetrasubstituted electron, a rich aromatic, an α,β - unsaturated ketone, an ester, four aliphatic groups and a single methyl group. The assignment is further supported by comparison of NMR data to literature NMR data (Hassan, 2007; Aly et al., 2010).

4.6 ^{1}H-NMR data table for dehydrocurvularin obtained in MeOD

H number*	δ_H (ppm) - (multiplicity, coupling (Hz), integral)	δ_H (ppm) - (multiplicity, coupling (Hz), integral) (reported in Hassan, 2007)
1		
2A	3.74 (d, J = 16.6Hz, 1H)	3.72 (d, J = 16.6Hz, 1H)
2B	3.46 (d, J = 16.6Hz, 1H)	3.44 (d, J = 16.6Hz, 1H)
3		
4	6.30 (d, J = 2.3Hz, 1H)	6.28 (d, J = 2.0Hz, 1H)
5		
6	6.26 (d, J = 2.3Hz, 1H)	6.24 (d, J = 2.0Hz, 1H)
7		
8		
9		
10	6.51 (dt, J = 15.6, 1.1Hz, 1H)	6.49 (d, J = 15.4Hz, 1H)
11	6.59 (ddd, J = 15.5, 8.4, 5.7Hz, 1H)	6.57 (ddd, J = 15.4, 8.2, 5.6Hz, 1H)
12A	2.41 (ddddd, *J* = 14.7, 7.4, 5.8, 2.7, 1.4 Hz, 1H)	2.38 (m, 1H)
12B	2.36-2.26 (m, 1H)	2.29 (m, 1H)
13A	2.01-1.93 (m, 1H)	1.96 (m, 1H)
13B	1.64-1.55 (m, 2H)**	1.56 (m, 1H)
14A	1.91-1.81 (m, 1H)	1.85 (m, 1H)
14B	1.64-1.55 (m, 2H)**	1.58 (m, 1H)
15	4.82 (tdd, J = 12.8, 6.2, 4.2Hz, 1H)	4.82 (m, 1H)
CH_3	1.20 (d, J=6.4Hz, 3H)	1.18 (d, J=6.6Hz, 3H)

* = Numbering of atoms is in accordance with literature (Hassan, 2007) and SI-1.3.4.

** = two peaks are indistinguishable from one another so are integrated together.

1H belongs to 13B (closed to 1.55) and the other 1H belongs to 14B (closer to 1.64).

4.7 ^{13}C-NMR data table for dehydrocurvularin obtained in MeOD

C number*	δc (ppm)	δc (ppm) (reported in Hassan, 2007)
1	173	173
2	42.4	42.4
3	137	137
4	112.2	112.2
5	162	162
6	102.8	102.8
7	162.3	162.2
8	117.9	117.8
9	200.1	200
10	133.4	133.3
11	154.4	154.3
12	34.2	34.2
13	25.5	25.4
14	35.2	35.1
15	74.3	74.2
CH_3	20.4	20.3

* = Numbering of atoms is in accordance with literature (Hassan, 2007) and SI-1.3.4.

4.8 ^{1}H-NMR spectrum of dehydrocurvularin in MeOD (Bruker Avance HD 600, at 600 MHZ)structure elucidation

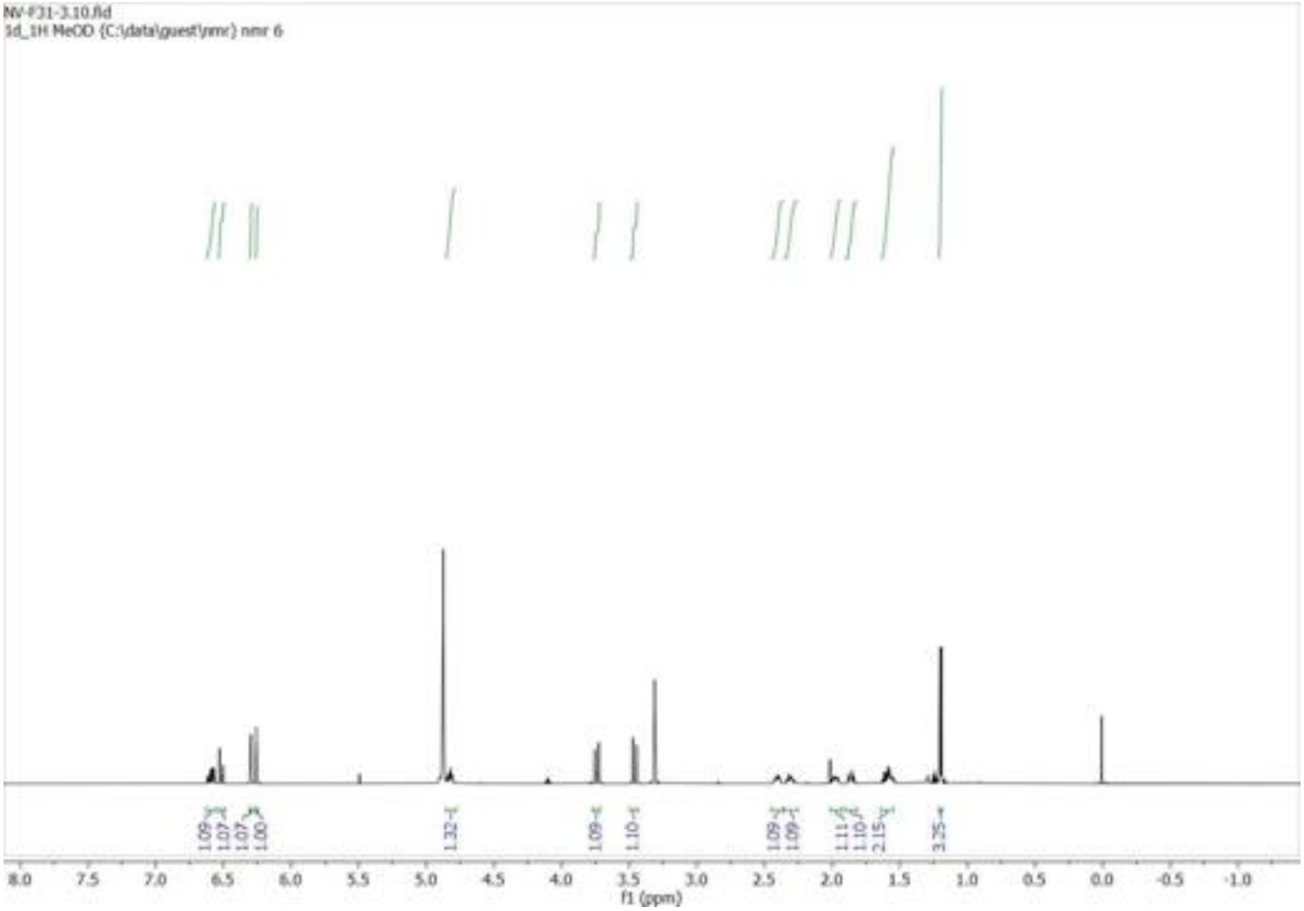

4.9 ^{13}C-NMR spectrum of dehydrocurvularin in MeOD (Bruker Avance HD 600, at 600 MHZ)structure elucidation

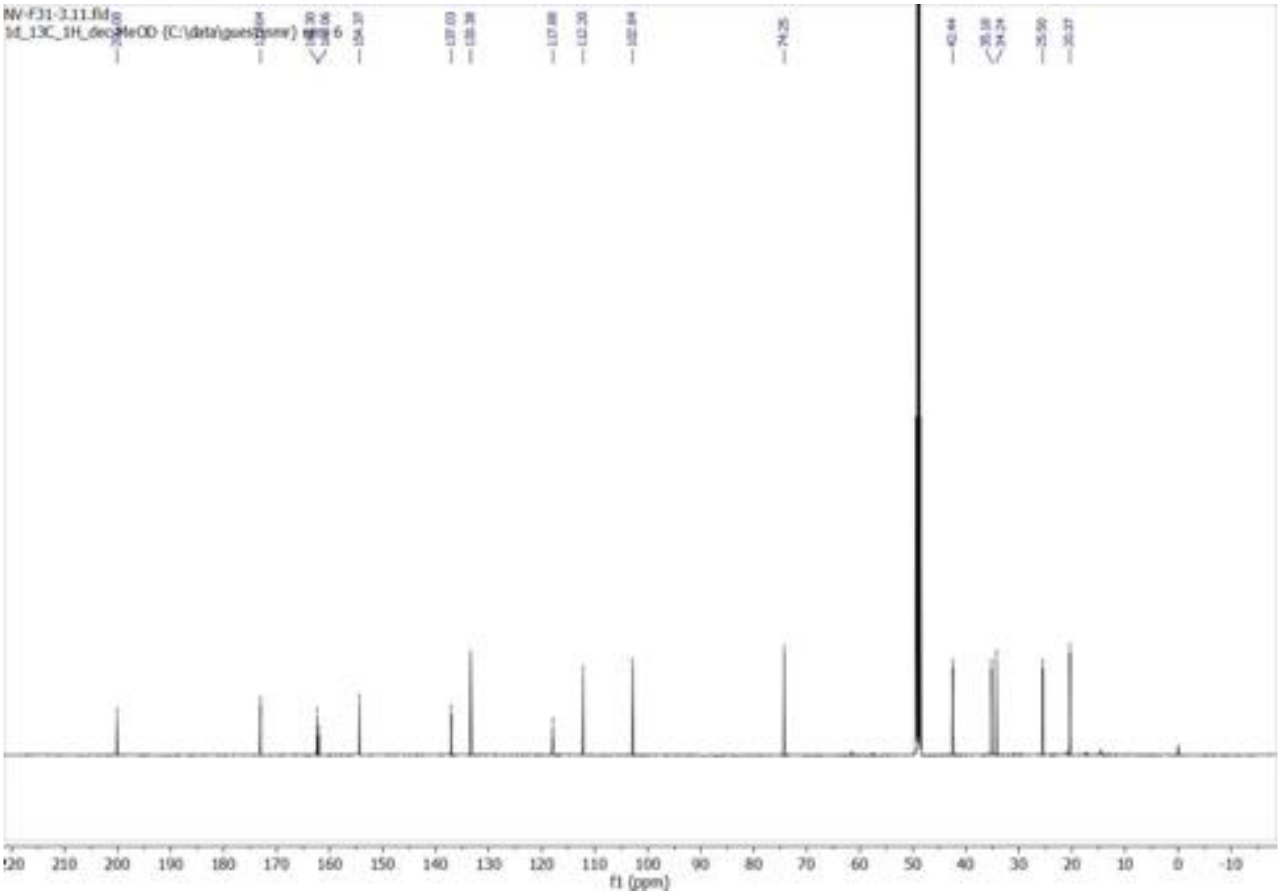

4.10 HSQC spectrum of dehydrocurvularin in MeOD (Bruker Avance HD 600) – ^{1}H-NMR displayed on the x-axis and ^{13}C-NMR on the y-axis.

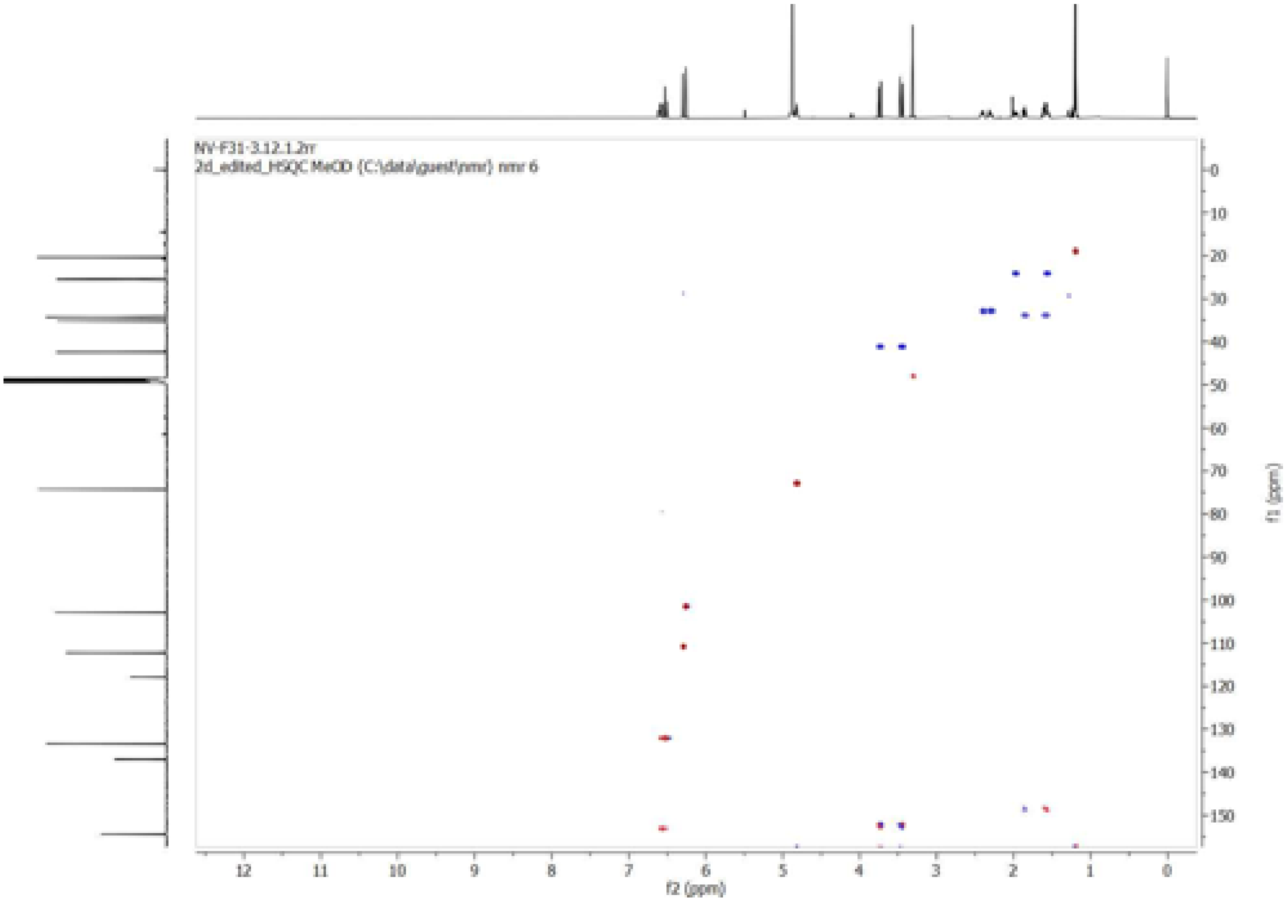

5 Rational for MS^n structural characterization of sumalarin C

The mass feature with a *m/z* of 413.1254 was identified to show similar characteristics as dehydrocurvularin after metabolomics processing with MZmine 2. After iterative attempts to purify *m/z* 413.1254 from large scale cultures using preparative thin layer chromatography and semi-preparative HPLC, this compound could not be completely isolated from dehydrocurvularin and other degradation products in a sufficient quantity for NMR characterization. UPLC-HRMS/MS scans were compared to freely available libraries of experimentally derived MS^2 data via the GNPS online interface (version 28.2) and *m/z* 413.1254 could tentatively be assigned as sumalarin C based on a molecular formula of $C_{19}H_{25}O_8S$. To further increase the confidence in the assignment of sumalarin C annotation, tandem MS (MS^n) spectra were acquired. Dehydrocurvularin and sumalarin C are structurally similar, with sumalarin C containing an additional 2-hydroxy-3-mercaptopropanoic acid appended at C11 of the curvularin macrocycle (SI-4.1).

Comparison of MS^2 spectra of dehydrocurvularin and sumalarin C contained similar fragment ions (SI-5.2 and SI-5.4) that indicates a high degree of structural similarity between the two compounds. Table SI-5.4 gives proposed fragment ions structures found in tandem MS data where each were

observed below 10 ppm error. Key fragment ions, *m/z* 291.12 and 273.11, 169.05, 167.03, 123.08, 95.09 and 81.07 are observed in the MS^2 spectra of both sumalarin C and dehydrocurvularin suggesting similar structural features. Critically, the fragment ion *m/z* 291.12 is produced by the loss of 2-hydroxy-3-mercaptopropanoic acid to regenerate dehydrocurvularin (see SI-5.2: DHC-$[M+H]^+$ and DHC-$[M+H-H_2O]^+$). Furthermore, a fragment ion with a *m/z* of 245.08 was uniquely observed in the MS^2 spectrum of sumalarin C, and further activation ($MS^2 \rightarrow MS^3$) of this fragment ion produced a characteristic loss of 2-hydroxy-3-mercaptopropanoic acid (*m/z* 245 → *m/z* 123) as well as hydrocarbon fragments $C_8H_{11}O^+$, $C_7H_{11}^+$, and $C_6H_9^+$ that can be found in both MS^2 spectra of sumalarin C and dehydrocurvularin (SI-5.2 and SI-5.3). From these data, we are confident in the annotation of the *m/z* 413.13 mass feature to be sumalarin C.

5.1 Molecular structure of sumalarin C.

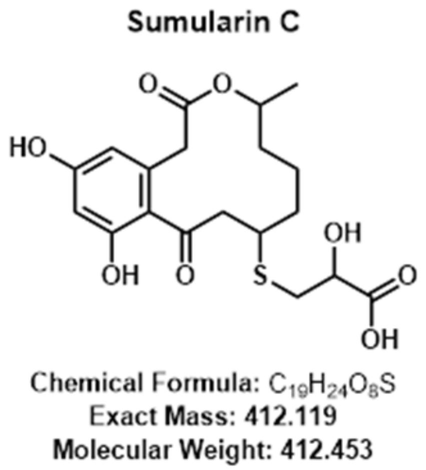

5.2 MS^2 spectrum of sumalarin C (SumC) $[M+H]^+$ ion (*m/z* 413.1254).

Asterisks indicate fragment ions also found in MS2 spectra of dehydrocurvularin (DHC) [M+H]+ ion (m/z 291.1216), Red asterisks denote fragment ions presented in SI-5.4.

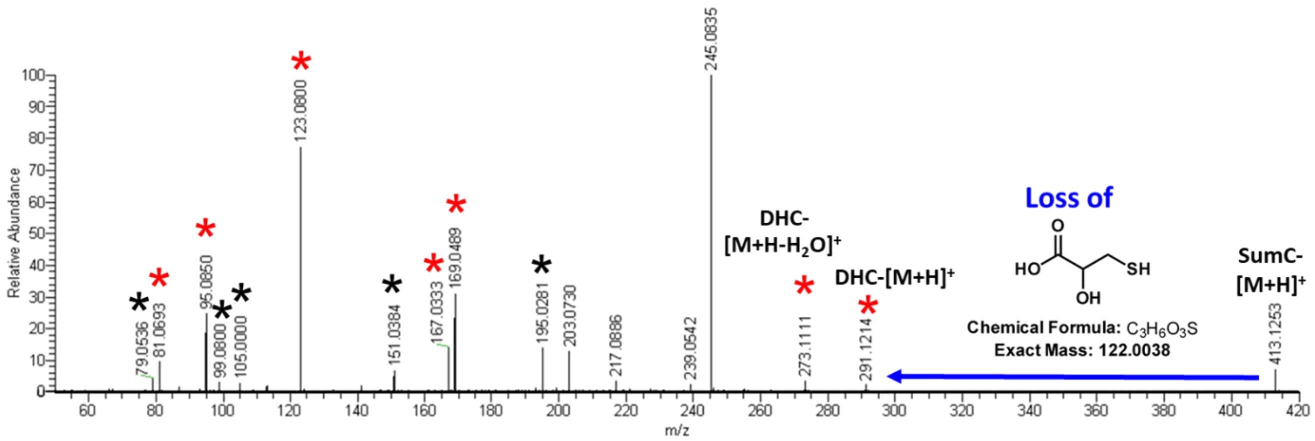

5.3 MS^3 spectrum of sumalarin C MS^2 [M+H]$^+$ critical fragment ion (*m/z* 245.0833).

Neutral loss confirms the presence of 2-hydroxy-3-mercaptopropanoic acid. Low mass range fragment ions are consistent with those observed from MS^2 spectra of dehydrocurvularin and sumalarin C (presented in SI-5.2 and SI-5.4).

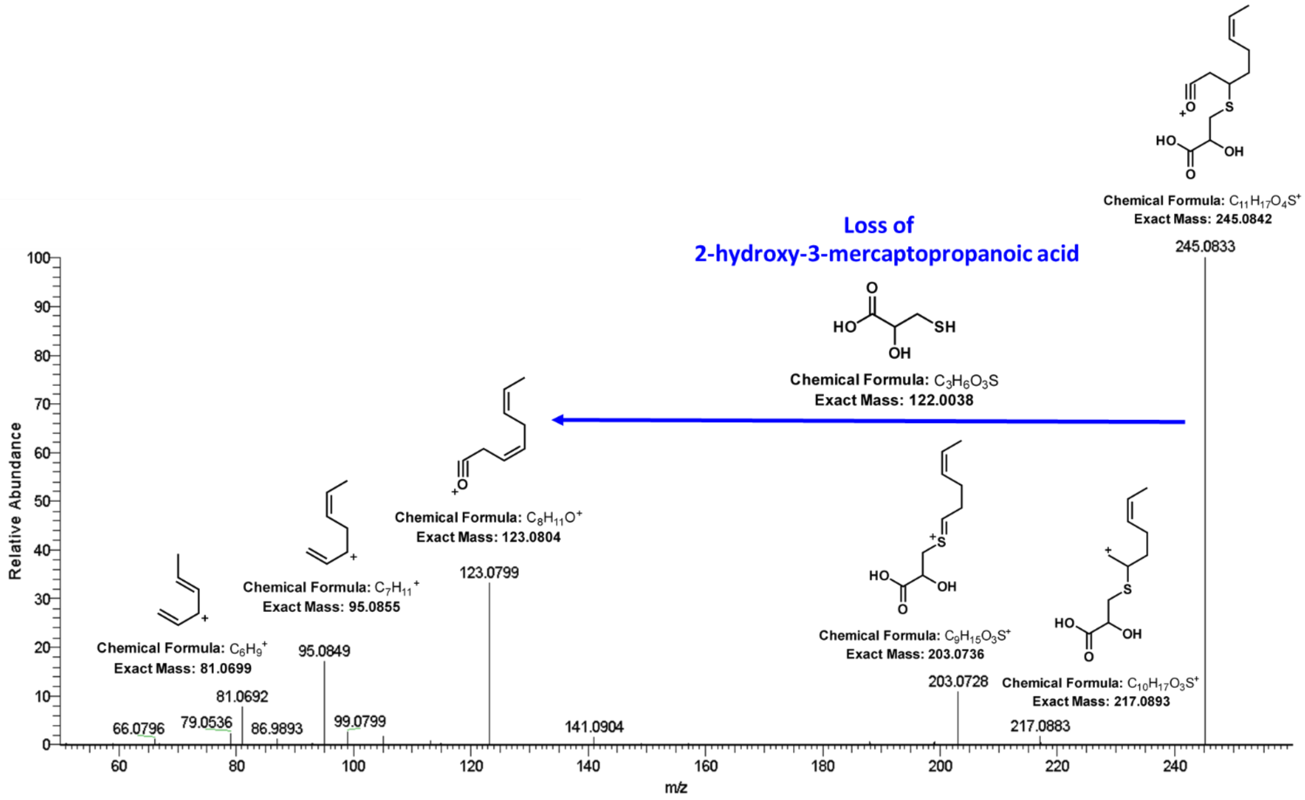

5.4 Table of proposed fragment structures observed from sumalarin C (SumC) and dehydrocurvularin (DHC).

Proposed Fragment Structure	Calculated *m/z*	Experimental m/z (DHC)	Experimental m/z (SumC)
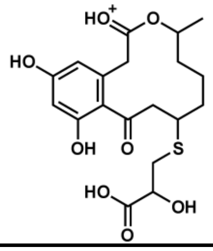	413.1265	n/a	413.1253 (-2.9 ppm)
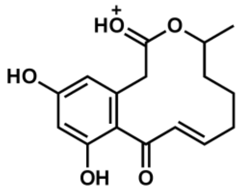	291.1227	291.1217 (-3.4 ppm)	291.1217 (-3.4 ppm)
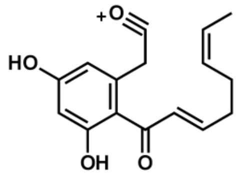	273.1121	273.1113 (-2.9 ppm)	273.1113 (-2.9 ppm)
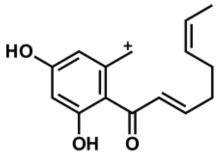	245.1172	245.1163 (-3.7 ppm)	n/a
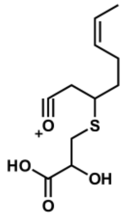	245.0842	n/a	245.0836 (-2.4 ppm)
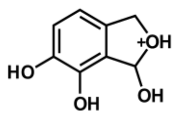	169.0495	169.0489 (-3.5 ppm)	169.0490 (-3.0 ppm)
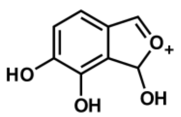	167.0339	167.0333 (-3.6 ppm)	167.0334 (-3.0 ppm)
$C_8H_{11}O^+$	123.0804	123.0799 (-4.1 ppm)	123.0800 (-3.2 ppm)
$C_7H_{11}^+$	95.0855	95.0850 (-5.3 ppm)	95.0850 (-5.3 ppm)
$C_6H_9^+$	81.0699	81.0693 (-7.4 ppm)	81.0693 (-7.4 ppm)

6 Annotation of UPLC-HRMS mass features based on tandem MS data

6.1 Table of secondary metabolite mass feature MS^2 annotations based on GNPS and Massbank spectral library matches (cosine scores) and SIRIUS/CSI-FingerID *in silico* fragmentation analysis rankings (% score).

Secondary Metabolite	GNPS library match (cosine score)	CSI FINGER-ID rank (% score)
11-hydroxycurvularin	-	1 (97.80)
11-methoxycurvularin	-	1 (96.45)
3-aipta	-	1 (51.15)
altenuene	Yes (0.94)	1 (88.52)
altenusin	-	1 (77.73)
alternariol	-	1 (96.92)
alternariol monomethyl ether	-	1 (90.74)
altersetin	-	1 (92.83)
curvularin	Yes (0.81)	1 (98.13)
cyclosulfoxicurvularin	-	1 (80.43)
cyclothiocurvularin	-	1 (68.09)
dehydrocurvularin (DHC)	Yes (0.74)	1 (85.36)
dihydrotentoxin	-	1 (80.00)
sulfoxicurvularin	-	not in structural databases
sumalarin A	-	1 (66.23)
sumalarin C	Yes (0.86)	1 (97.55)
tentoxin	-	1 (62.79)
tenuazonic acid	Yes (0.78)	1 (93.90)
tricycloalternarene 2b	Yes (0.73)	2 (73.06)

6.2 Mirror plots comparing top database spectral matches to MS^2 spectra from mass features annotated in this study.

Comparisons performed as part of GNPS FBMN workflow. Top spectra (black) are from the present study, bottom spectra (green) are from online databases.

A) Curvularin

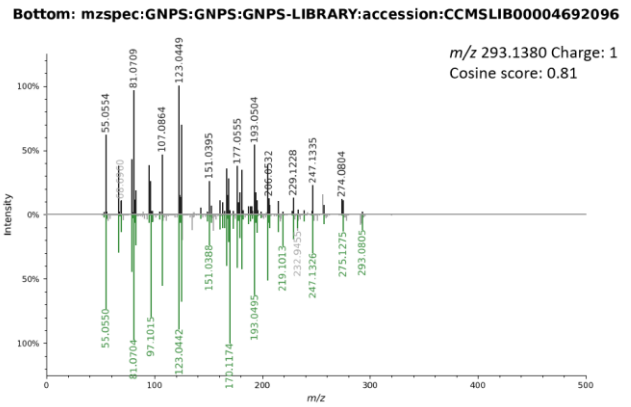

B) Sumalarin C

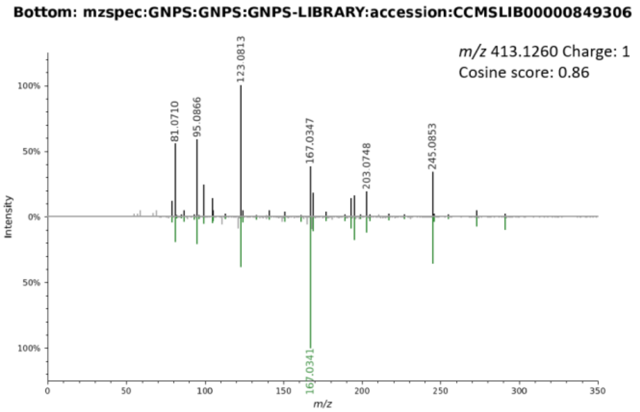

C) Dehydrocurvularin

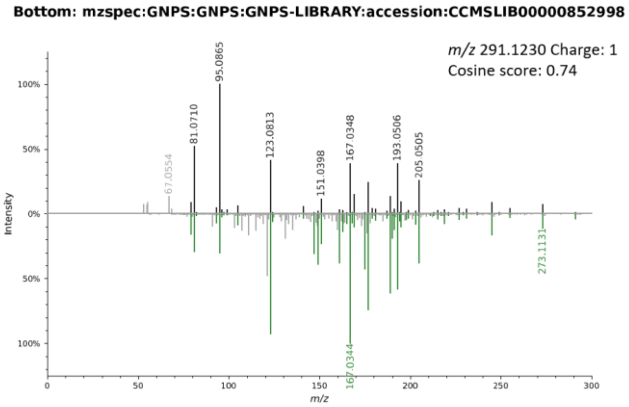

D) Tenuazonic acid

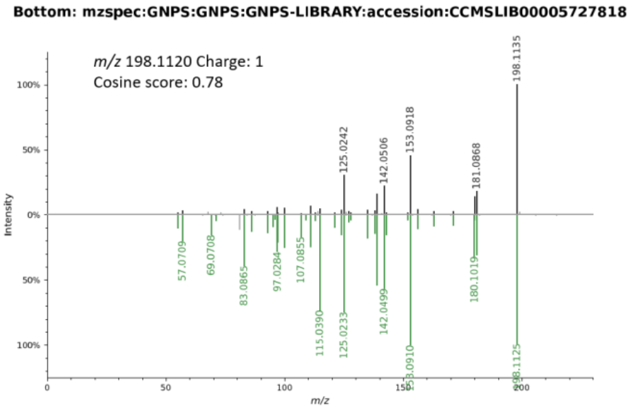

E) Tricycloalternarene 2b

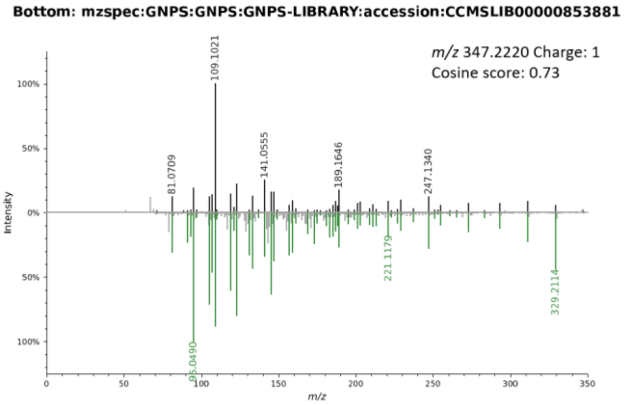

F) Altenuene

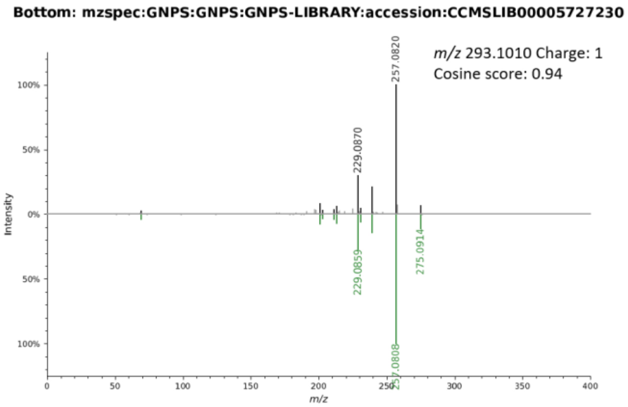

7 Summary statistics for whole-genome assemblies from Illumina short-read sequences for dehydrocurvularin producing *Alternaria* strains.

Isolate name	DET2035	KAS5321	KAS5743
Species	*A. arborescens*	*A. arborescens*	*A. alternata*
Fold sequence coverage	135.4	115.8	124.2
Contig number (>500 bp)	163	125	211
N50 (kb)	671.5	667.2	1354.8
L50 (contigs)	17	14	10
Length (Mb)	33.10	33.00	34.11
GC%	51.1	51.1	51.1
Complete BUSCO genes (%)	99.3	99.3	99.1
Missing BUSCO genes (%)	0.7	0.7	0.8
NCBI accession	JAERPI000000000	JAERNY000000000	JAERND000000000

8 Results returned with greater than 80% sequence similarity from BLASTn search of the full NCBI nt database using each of the four core *A. arborescens* DET2035 *dhc* gene sequences as queries.

Taxon	Strain	Query locus	Hit locus	Query Cover	% Identity	Accession
Alternaria atra	CS162	*dhc2*	LOCUS59, mRNA	93%	98.8	XM_043307652.1
		dhc3	LOCUS61, mRNA	83%	97.0	XM_043307654.1
		dhc4	LOCUS62, mRNA	93%	98.2	XM_043307655.1
		dhc5	LOCUS63, mRNA	98%	98.5	XM_043307656.1
Alternaria solani	NL03003	*dhc2*	chromosome 8, complete sequence	100%	94.9	CP022031.1
		dhc3	chromosome 8, complete sequence	100%	95.2	CP022031.1
		dhc4	chromosome 8, complete sequence	100%	95.4	CP022031.1
		dhc5	chromosome 8, complete sequence	99%	95.0	CP022031.1
Pyrenophora tritici-repentis	Pt-1C-BFP	*dhc2*	MFS gliotoxin efflux transporter GliA, mRNA	92%	90.5	XM_001934686.1
		dhc3	phenolpthiocerol synthesis polyketide synthase ppsB, mRNA	96%	86.3	XM_001934685.1
		dhc4	C6 zinc finger domain containing protein, mRNA	96%	88.0	XM_001934684.1
		dhc5	conidial yellow pigment biosynthesis polyketide synthase, mRNA	93%	88.2	XM_001934683.1

*see Manuscript Table 3 for *A. cinerariae* genes

9 List of whole-genome assemblies of 281 Pleosporalean fungi from various public sources.

Species	Strain	Source	Accession	Family
Aaosphaeria arxii	CBS 175.79	NCBI	GCF_010015735.1	Dacampiaceae (?)
Aaosphaeria arxii	FJI-L9-BK-P1	NCBI	GCA_020086995.1	Dacampiaceae (?)
Alternaria alstroemeriae	CBS 118809	NCBI	GCA_020284155.1	Pleosporaceae
Alternaria alternata	ATCC11680	JGI	mycocosm.jgi.doe.gov; Alalt1	Pleosporaceae
Alternaria alternata	ATCC34957	NCBI	GCA_001443195.2	Pleosporaceae
Alternaria alternata	ATCC66891	JGI	mycocosm.jgi.doe.gov; Alalte1	Pleosporaceae
Alternaria alternata	B2a	NCBI	GCA_001696825.1	Pleosporaceae
Alternaria alternata	B3	NCBI	GCA_014154925.1	Pleosporaceae
Alternaria alternata	BMP0270	JGI	mycocosm.jgi.doe.gov; Aalte1	Pleosporaceae
Alternaria alternata	DET2001	NCBI	GCA_020736775.1	Pleosporaceae
Alternaria alternata	DET2010	NCBI	GCA_020736745.1	Pleosporaceae
Alternaria alternata	DET2019	NCBI	GCA_020736755.1	Pleosporaceae
Alternaria alternata	EV-MIL-31	NCBI	GCA_016097525.1	Pleosporaceae
Alternaria alternata	FERA1177	NCBI	GCA_004154755.1	Pleosporaceae
Alternaria alternata	hznu325	NCBI	GCA_020449105.1	Pleosporaceae
Alternaria alternata	JS-0527	NCBI	GCA_011420255.1	Pleosporaceae
Alternaria alternata	JS-1623	NCBI	GCA_009650635.1	Pleosporaceae
Alternaria alternata	KAS5516	NCBI	GCA_020736765.1	Pleosporaceae
Alternaria alternata	MOD1-FUNGI5	NCBI	GCA_004634295.1	Pleosporaceae
Alternaria alternata	MPI-PUGE-AT-0064	JGI	mycocosm.jgi.doe.gov; Altalt1	Pleosporaceae
Alternaria alternata	NAP07	NCBI	GCA_009932595.1	Pleosporaceae
Alternaria alternata	PF1	NCBI	GCA_018104255.1	Pleosporaceae
Alternaria alternata	PN1	NCBI	GCA_011420445.1	Pleosporaceae
Alternaria alternata	PN2	NCBI	GCA_011420565.1	Pleosporaceae
Alternaria alternata	SRC1lrK2f	NCBI	GCA_001642055.1	Pleosporaceae
Alternaria alternata	Y784-BC03	NCBI	GCA_020085065.1	Pleosporaceae
Alternaria alternata	Z7	NCBI	GCA_014751505.1	Pleosporaceae
Alternaria alternata (A. citriarbusti)	BMP2343	JGI	mycocosm.jgi.doe.gov; Altci1	Pleosporaceae
Alternaria alternata (A. limoniasperae)	BMP2335	JGI	mycocosm.jgi.doe.gov; Altli1	Pleosporaceae
Alternaria alternata (A. mali)	BMP3064	JGI	mycocosm.jgi.doe.gov; Amal3064	Pleosporaceae
Alternaria alternata (A. tenuissima)	ANJ	NCBI	GCA_017589455.1	Pleosporaceae
Alternaria alternata (A. tenuissima)	BMP0304	JGI	mycocosm.jgi.doe.gov; Altte1	Pleosporaceae
Alternaria alternata (A. tenuissima)	FERA1082	NCBI	GCA_004154745.1	Pleosporaceae
Alternaria alternata (A. tenuissima)	FERA1164	NCBI	GCA_004156015.1	Pleosporaceae
Alternaria alternata (A. tenuissima)	FERA1166	NCBI	GCA_004156035.1	Pleosporaceae
Alternaria alternata (A. tenuissima)	FERA24350	NCBI	GCA_004154735.1	Pleosporaceae
Alternaria alternata (A. tenuissima)	FERA635	NCBI	GCA_004168565.1	Pleosporaceae
Alternaria alternata (A. tenuissima)	FERA648	NCBI	GCA_004154765.1	Pleosporaceae
Alternaria alternata (A. tenuissima)	FERA743	NCBI	GCA_004154845.1	Pleosporaceae
Alternaria arborescens	BMP0308	JGI	mycocosm.jgi.doe.gov; Altar1	Pleosporaceae
Alternaria arborescens	DET2008	NCBI	GCA_020736785.1	Pleosporaceae
Alternaria arborescens	EGS39-128	NCBI	GCA_000256225.1	Pleosporaceae
Alternaria arborescens	FERA675	NCBI	GCA_004154835.1	Pleosporaceae
Alternaria arborescens	MOD1-FUNGI6	NCBI	GCA_004634205.1	Pleosporaceae
Alternaria arborescens	NRRL20593	NCBI	GCA_013282825.1	Pleosporaceae
Alternaria arborescens	RGR97.0013	NCBI	GCA_004155955.1	Pleosporaceae
Alternaria arborescens	RGR97.0016	NCBI	GCA_004154815.1	Pleosporaceae

Species	Strain	Source	Accession	Family
Alternaria arborescens (A. mali)	BMP3063	JGI	mycocosm.jgi.doe.gov; Amal3063	Pleosporaceae
Alternaria atra	CS162	NCBI	GCA_907166805.1	Pleosporaceae
Alternaria atra	MOD1-FUNGI7	NCBI	GCA_004634305.1	Pleosporaceae
Alternaria brassicae	J3	NCBI	GCA_004936725.1	Pleosporaceae
Alternaria brassicicola	Abra43	NCBI	GCA_002796735.1	Pleosporaceae
Alternaria brassicicola	Altbr1	JGI	mycocosm.jgi.doe.gov; Altbr1	Pleosporaceae
Alternaria brassicicola	ATCC96836	NCBI	GCA_000174375.1	Pleosporaceae
Alternaria burnsii	CBS107.38	NCBI	GCA_013036055.1	Pleosporaceae
Alternaria capsici	BMP0180	JGI	mycocosm.jgi.doe.gov; Altca1	Pleosporaceae
Alternaria carthami	BMP1963	JGI	mycocosm.jgi.doe.gov; Altcar1	Pleosporaceae
Alternaria consortialis	JCM1940	NCBI	GCA_001950455.1	Pleosporaceae
Alternaria crassa	BMP0172	JGI	mycocosm.jgi.doe.gov; Altcr1	Pleosporaceae
Alternaria dauci	BMP0167	JGI	mycocosm.jgi.doe.gov; Altda1	Pleosporaceae
Alternaria gaisen	BMP2338	JGI	mycocosm.jgi.doe.gov; Altga1	Pleosporaceae
Alternaria gaisen	FERA650	NCBI	GCA_004156025.2	Pleosporaceae
Alternaria gaisen (A. fragaria)	BMP3062	JGI	mycocosm.jgi.doe.gov; Altfr1	Pleosporaceae
Alternaria gansuensis	LYZ1412	NCBI	GCA_009289805.1	Pleosporaceae
Alternaria longipes	BMP0313	JGI	mycocosm.jgi.doe.gov; Altlo1	Pleosporaceae
Alternaria longipes	CBS540.94	NCBI	GCA_019059555.1	Pleosporaceae
Alternaria macrospora	BMP1949	JGI	mycocosm.jgi.doe.gov; Altma1	Pleosporaceae
Alternaria panax	BNCC115425	NCBI	GCA_019702505.1	Pleosporaceae
Alternaria porri	BMP0178	JGI	mycocosm.jgi.doe.gov; Altpo1	Pleosporaceae
Alternaria rosae	MPI-PUGE-AT-0040	JGI	mycocosm.jgi.doe.gov; Altro1	Pleosporaceae
Alternaria solani	BMP0185	JGI	mycocosm.jgi.doe.gov; Altso1	Pleosporaceae
Alternaria solani	HWC-168-2012p	NCBI	GCA_002837235.1	Pleosporaceae
Alternaria solani	NL03003	NCBI	GCA_002952155.1	Pleosporaceae
Alternaria sp.	MG1	NCBI	GCA_003574525.1	Pleosporaceae
Alternaria tangelonis	BMP2327	JGI	mycocosm.jgi.doe.gov; Altta1	Pleosporaceae
Alternaria tomatophila	BMP2032	JGI	mycocosm.jgi.doe.gov; Alttom1	Pleosporaceae
Amniculicola lignicola	CBS 123094	NCBI	GCA_010015725.1	Amniculicolaceae
Ampelomyces quisqualis	BRIP 72107	NCBI	GCA_018398575.1	Leptosphaeriaceae (Phaeosphaeriaceae?)
Ampelomyces quisqualis	HMLAC05119	NCBI	GCA_010094095.1	Leptosphaeriaceae (Phaeosphaeriaceae?)
Ascochyta fabae	247/15	NCBI	GCA_004335285.1	Didymellaceae
Ascochyta koolunga	CND-11-149	NCBI	GCA_004151575.1	Didymellaceae
Ascochyta koolunga	GRP-15-464	NCBI	GCA_004151145.1	Didymellaceae
Ascochyta koolunga	PIN-15-415	NCBI	GCA_004151165.1	Didymellaceae
Ascochyta lentis	Al4	NCBI	GCA_004011705.1	Didymellaceae
Ascochyta lentis	F16132	NCBI	GCA_004335125.1	Didymellaceae
Ascochyta lentis	F16293-3	NCBI	GCA_004335145.1	Didymellaceae
Ascochyta lentis	FT16116	NCBI	GCA_004335135.1	Didymellaceae
Ascochyta lentis	JD202.22	NCBI	GCA_016097305.1	Didymellaceae
Ascochyta lentis	JD202.9	NCBI	GCA_016097315.1	Didymellaceae
Ascochyta lentis	JD202.ect	NCBI	GCA_016097295.1	Didymellaceae
Ascochyta lentis	Kewell	NCBI	GCA_016097375.1	Didymellaceae

Species	Strain	Source	Accession	Family
Ascochyta rabiei	ArDII	NCBI	GCA_001630375.1	Didymellaceae
Ascochyta rabiei	Me14	NCBI	GCF_004011695.1	Didymellaceae
Ascochyta viciae	FOR-16-616	NCBI	GCA_004335155.1	Didymellaceae
Ascochyta viciae-villosae	ONG-16-641	NCBI	GCA_004335205.1	Didymellaceae
Beverwykella pulmonaria	JCM 9230	NCBI	GCA_001599595.1	Melanommataceae (?)
Bimuria novae-zelandiae	CBS 107.79	NCBI	GCA_010015655.1	Didymosphaeriaceae
Bipolaris cookei	LSLP18.3	NCBI	GCA_002286855.1	Pleosporaceae
Bipolaris maydis	ATCC48331	NCBI	GCA_000354255.1	Pleosporaceae
Bipolaris maydis	BM1	NCBI	GCA_019454015.1	Pleosporaceae
Bipolaris maydis	C5	NCBI	GCA_000338975.1	Pleosporaceae
Bipolaris maydis (Cochliobolus heterostrophus)	C4-1	JGI	mycocosm.jgi.doe.gov; CocheC4-1	Pleosporaceae
Bipolaris maydis (Cochliobolus heterostrophus)	Hm540-1	JGI	mycocosm.jgi.doe.gov; ChetHm540-1	Pleosporaceae
Bipolaris oryzae	ATCC44560	NCBI	GCA_000523455.1	Pleosporaceae
Bipolaris oryzae	TG12bL2	NCBI	GCA_001675385.1	Pleosporaceae
Bipolaris sorokiniana	BRIP10943a	NCBI	GCA_008452735.1	Pleosporaceae
Bipolaris sorokiniana	BRIP27492a	NCBI	GCA_008452725.1	Pleosporaceae
Bipolaris sorokiniana	BS112	NCBI	GCA_004329375.1	Pleosporaceae
Bipolaris sorokiniana	Gansu	NCBI	GCA_013416765.1	Pleosporaceae
Bipolaris sorokiniana	ND90Pr	NCBI	GCA_000338995.1	Pleosporaceae
Bipolaris sorokiniana	Shoemaker	NCBI	GCA_013416765.1	Pleosporaceae
Bipolaris sorokiniana	WAI2406	NCBI	GCA_008452705.1	Pleosporaceae
Bipolaris sorokiniana	WAI2411	NCBI	GCA_008452715.1	Pleosporaceae
Bipolaris victoriae	FI3	NCBI	GCA_000527765.2	Pleosporaceae
Bipolaris zeicola	26-R-13	NCBI	GCA_000523435.1	Pleosporaceae
Bipolaris zeicola	GZL1	NCBI	GCA_016906865.1	Pleosporaceae
Boeremia exigua	MPI-SDFR-AT-0100	NCBI	GCA_020726555.1	Didymellaceae
Byssothecium circinans	CBS 675.92	NCBI	GCA_010015675.1	Massarinaceae (Dacampiaceae?)
Clathrospora elynae	CBS 161.51	NCBI	GCA_010015635.1	Diademaceae
Clavariopsis aquatica	WD(A)-00-1	NCBI	GCA_013620735.1	?
Clohesyomyces aquaticus	CBS 115471	NCBI	GCA_002105025.1	Lindgomycetaceae
Corynespora cassiicola	C7	NCBI	GCA_900169545.1	Corynesporascaceae
Corynespora cassiicola	CAL-4	NCBI	GCA_006523515.1	Corynesporascaceae
Corynespora cassiicola	CBS129.25	NCBI	GCA_002976015.1	Corynesporascaceae
Corynespora cassiicola	CC_29	NCBI	GCA_019202905.1	Corynesporascaceae
Corynespora cassiicola	CC01	NCBI	GCA_016906425.1	Corynesporascaceae
Corynespora cassiicola	CSRI1	NCBI	GCA_002975815.1	Corynesporascaceae
Corynespora cassiicola	LPO7	NCBI	GCA_002975495.1	Corynesporascaceae
Corynespora cassiicola	Philippines	NCBI	GCA_003016335.1	Corynesporascaceae
Corynespora cassiicola	TCl3	NCBI	GCA_006519745.1	Corynesporascaceae
Corynespora cassiicola	UM 591	NCBI	GCA_000603925.1	Corynesporascaceae
Corynespora olivacea	CBS 114450	NCBI	GCA_019202945.1	Corynesporascaceae
Cucurbitaria berberidis	CBS 394.84	NCBI	GCF_010015615.1	Cucurbitariaceae
Curvularia eragrostidis	C52	NCBI	GCA_020744315.1	Pleosporaceae
Curvularia geniculata	P1	NCBI	GCA_016162275.1	Pleosporaceae
Curvularia geniculata	W3	NCBI	GCA_002982235.1	Pleosporaceae
Curvularia lunata	CX-3	NCBI	GCA_000743335.1	Pleosporaceae
Curvularia lunata	W3	NCBI	GCA_005212705.1	Pleosporaceae
Curvularia lunata (Cochliobolus lunatus)	m118	JGI	Coclu2	Pleosporaceae
Curvularia papendorfii	UM_226	NCBI	GCA_000817285.1	Pleosporaceae
Curvularia sp	IFB-Z10	NCBI	GCA_002161795.1	Pleosporaceae
Decorospora gaudefroyi	Decga1	JGI	Decga1	Pleosporaceae
Decorospora gaudefroyi	P77	NCBI	GCA_010015605.1	Pleosporaceae
Delitschia confertaspora	ATCC 74209	NCBI	GCA_010093945.1	Delitschiaceae
Dendryphion nanum	MPI-CAGE-CH-0243	NCBI	GCA_020744415.1	Torulaceae
Didymella arachidicola	YY187	NCBI	GCA_016630955.1	Didymellaceae
Didymella exigua	CBS 183.55	NCBI	GCF_010094145.1	Didymellaceae
Didymella heteroderae	28M1	NCBI	GCA_011058895.1	Didymellaceae

Species	Strain	Source	Accession	Family
Didymella keratinophila	9M1	NCBI	GCA_011058865.1	Didymellaceae
Didymella lethalis	FOR-16-620	NCBI	GCA_004335255.1	Didymellaceae
Didymella lethalis	GRM-16-623	NCBI	GCA_004335245.1	Didymellaceae
Didymella pinodes	BAL-11-01	NCBI	GCA_004151185.1	Didymellaceae
Didymella pinodes	GRP-11-30	NCBI	GCA_004151295.1	Didymellaceae
Didymella pinodes	RIV-11-185	NCBI	GCA_004151305.1	Didymellaceae
Didymella pinodes	WTN-11-157	NCBI	GCA_004151525.1	Didymellaceae
Didymella pinodes	YEL-11-87	NCBI	GCA_004151515.1	Didymellaceae
Didymella segeticola	GZSQ-4	NCBI	GCA_004522025.1	Didymellaceae
Didymosphaeria enalia	CBS 304.66	NCBI	GCA_010094045.1	Didymosphaeriaceae
Dothidotthia symphoricarpi	CBS 119687	NCBI	GCF_010015815.1	Dothidotthiaceae
Epicoccum latusicollum	T41	NCBI	GCA_015266435.1	Didymellaceae
Epicoccum nigrum	cf0051	NCBI	GCA_019721275.1	Didymellaceae
Epicoccum nigrum	ICMP 19927	NCBI	GCA_002116315.1	Didymellaceae
Epicoccum nigrum	P16	NCBI	GCA_009761125.1	Didymellaceae
Epicoccum sorghinum	BS2-1	NCBI	GCA_020272525.1	Didymellaceae
Epicoccum sorghinum	USPMTOX48	NCBI	GCA_001879705.1	Didymellaceae
Exserohilum rostratum	ER1	NCBI	GCA_019453395.1	Pleosporaceae
Exserohilum turcicum (Exserohilum turcica)	Et28A	NCBI	GCF_000359705.1	Pleosporaceae
Exserohilum turcicum (Setosphaeria turcica)	NY001	JGI	Settur3	Pleosporaceae
Helminthosporium solani	B-AC-16A	NCBI	GCA_000498615.1	Massarinaceae
Karstenula rhodostoma	CBS 690.94	NCBI	GCA_010093485.1	Didymosphaeriaceae
Laburnicola sp.	JP-R-44	NCBI	GCA_009805535.1	Didymosphaeriaceae
Laburnicola sp.	R22_1	NCBI	GCA_014281115.1	Didymosphaeriaceae
Lentithecium fluviatile	CBS 122367	NCBI	GCA_010405425.1	Lentitheciaceae
Leptosphaeria biglobosa	G12-14	NCBI	GCA_900465125.1	Leptosphaeriaceae
Leptosphaeria maculans	JN3	NCBI	GCF_000230375.1	Leptosphaeriaceae
Leptosphaeria maculans	NZT4	NCBI	GCA_900465115.1	Leptosphaeriaceae
Lindgomyces ingoldianus	ATCC 200398	NCBI	GCF_010093535.1	Lindgomycetaceae
Lophiostoma macrostomum	CBS 122681	NCBI	GCA_010405375.1	Lophiostomataceae
Lophiotrema nucula	CBS 627.86	NCBI	GCA_010015825.1	Lophiotremataceae
Macroventuria anomochaeta	CBS 525.71	NCBI	GCF_010093625.1	Didymellaceae
Massarina eburnea	CBS 473.64	NCBI	GCA_010093635.1	Massarinaceae
Massariosphaeria phaeospora	CBS 611.86	NCBI	GCA_011032825.1	Cyclothyriellaceae (?), Thyridariaceae (?)
Melanomma pulvis-pyrius	CBS 109.77	NCBI	GCA_010093585.1	Melanommataceae
Neoascochyta sp.	HWLR27	NCBI	GCA_019775935.1	Didymellaceae
Neocamarosporium betae	Pb1	NCBI	GCA_016612005.1	Pleosporaceae
Nigrograna mackinnonii	E5202H	NCBI	GCA_001007845.1	Nigrogranaceae
Ophiobolus disseminans	CBS 113818	NCBI	GCA_010093685.1	Leptosphaeriaceae (Phaeosphaeriaceae?)
Paracamarosporium sp.	OC-R06-R3	NCBI	GCA_019828545.1	Didymosphaeriaceae
Paradendryphiella salina	PS1_23737	NCBI	GCA_900634815.1	Pleosporaceae
Paraphaeosphaeria minitans	IMI134523	NCBI	GCA_015832175.1	Didymosphaeriaceae
Paraphaeosphaeria minitans	ZS-1	NCBI	GCA_009707825.1	Didymosphaeriaceae
Paraphaeosphaeria sporulosa	AP3s5-JAC2a	NCBI	GCF_001642045.1	Didymosphaeriaceae
Paraphoma chrysanthemicola	MPI-GEGE-AT-0034	NCBI	GCA_020744225.1	Phaeosphaeriaceae
Paraphoma chrysanthemicola	MPI-SDFR-AT-0120	NCBI	GCA_020744215.1	Phaeosphaeriaceae
Paraphoma sp.	B47-9	NCBI	GCA_001748405.1	Phaeosphaeriaceae
Parastagonospora avenae f. sp. avenae	Mt. Baker	NCBI	GCA_003501955.1	Phaeosphaeriaceae
Parastagonospora avenae f. sp. avenae	s258	NCBI	GCA_003501935.1	Phaeosphaeriaceae
Parastagonospora avenae f. sp. tritici	82-4841	NCBI	GCA_003503125.1	Phaeosphaeriaceae
Parastagonospora avenae f. sp. tritici	83-6011-2	NCBI	GCA_003502015.1	Phaeosphaeriaceae
Parastagonospora avenae f. sp. tritici	Hartney99	NCBI	GCA_003503195.1	Phaeosphaeriaceae
Parastagonospora avenae f. sp. tritici	IR10_5.2b	NCBI	GCA_003503205.1	Phaeosphaeriaceae
Parastagonospora avenae f. sp. tritici	Jansen 4_55	NCBI	GCA_003503165.1	Phaeosphaeriaceae
Parastagonospora avenae f. sp. tritici	SN11IR_2_1.1	NCBI	GCA_003502495.1	Phaeosphaeriaceae
Parastagonospora avenae f. sp. tritici	SN11IR_6_1.1	NCBI	GCA_003503115.1	Phaeosphaeriaceae

Species	Strain	Source	Accession	Family
Parastagonospora avenae f. sp. tritici	SN11IR_7_2.3	NCBI	GCA_003501975.1	Phaeosphaeriaceae
Parastagonospora nodorum	14FG141	NCBI	GCA_020581275.1	Phaeosphaeriaceae
Parastagonospora nodorum	15FG109	NCBI	GCA_020580655.1	Phaeosphaeriaceae
Parastagonospora nodorum	16FG166	NCBI	GCA_020577905.1	Phaeosphaeriaceae
Parastagonospora nodorum	FIN-2	NCBI	GCA_003497975.1	Phaeosphaeriaceae
Parastagonospora nodorum	LDN03-Sn4	NCBI	GCA_002267005.1	Phaeosphaeriaceae
Parastagonospora nodorum	Meck3	NCBI	GCA_020573915.1	Phaeosphaeriaceae
Parastagonospora nodorum	SN15	NCBI	GCF_000146915.1	Phaeosphaeriaceae
Parastagonospora nodorum	Sn2000	NCBI	GCA_002267045.1	Phaeosphaeriaceae
Parastagonospora nodorum	Sn79-1087	NCBI	GCA_002267025.1	Phaeosphaeriaceae
Parastagonospora nodorum	SWE-3	NCBI	GCA_003497965.1	Phaeosphaeriaceae
Periconia macrospinosa	DSE2036	NCBI	GCA_003073855.1	Periconiaceae
Phaeosphaeria sp.	A1 3.1a	NCBI	GCA_003501895.1	Phaeosphaeriaceae
Phaeosphaeria sp.	H6.2b	NCBI	GCA_003503105.1	Phaeosphaeriaceae
Phaeosphaeria sp.	MPI-PUGE-AT-0046c	NCBI	GCA_020747045.1	Phaeosphaeriaceae
Phoma herbarum	JCM 15942	NCBI	GCA_001599375.1	Didymellaceae
Phoma sp.	RAV-16-625	NCBI	GCA_004335185.1	Didymellaceae
Phoma sp.	XZ068	NCBI	GCA_004835665.1	Didymellaceae
Plenodomus tracheiphilus	IPT5	NCBI	GCA_010093695.1	Leptosphaeriaceae
Pleomassaria siparia	CBS 279.74	NCBI	GCA_010093715.1	Pleomassariaceae
Pleosporales sp.	UM 1110	NCBI	GCA_000263175.2	?
Polyplosphaeria fusca	CBS 125425	NCBI	GCA_010093805.1	Tetraplosphaeriaceae
Preussia sp.	BSL10	NCBI	GCA_001553865.1	Sporormiaceae
Pseudomonodictys sp.	A73	NCBI	GCA_019022785.1	Macrodiplodiopsidaceae
Pseudopyrenochaeta lycopersici	CRA-PAV_ER 1211	NCBI	GCA_000601435.1	Pseudopyrenochaetaceae
Pseudopyrenochaeta lycopersici	CRA-PAV_ER 1518	NCBI	GCA_003313425.1	Pseudopyrenochaetaceae
Pyrenochaeta sp.	DS3sAY3a	NCBI	GCA_001644535.1	Cucurbitariaceae
Pyrenochaeta sp.	MPI-SDFR-AT-0127	NCBI	GCA_020747015.1	Cucurbitariaceae
Pyrenochaeta sp.	UM 256	NCBI	GCA_000359685.2	Cucurbitariaceae
Pyrenophora graminea	CBS336.29	NCBI	GCA_012365135.1	Pleosporaceae
Pyrenophora seminiperda	CCB06	NCBI	GCA_000465215.2	Pleosporaceae
Pyrenophora teres f. maculata	DEN2.6	NCBI	GCA_014334755.1	Pleosporaceae
Pyrenophora teres f. maculata	FGOB10Ptm-1	NCBI	GCA_014334795.1	Pleosporaceae
Pyrenophora teres f. maculata	NZKF2	NCBI	GCA_014334775.1	Pleosporaceae
Pyrenophora teres f. maculata	P-A14	NCBI	GCA_014334815.1	Pleosporaceae
Pyrenophora teres f. maculata	SG1	NCBI	GCA_900231935.2	Pleosporaceae
Pyrenophora teres f. maculata (?)	LQA	NCBI	GCA_020027095.1	Pleosporaceae
Pyrenophora teres f. teres	0-1	NCBI	GCA_006112615.1	Pleosporaceae
Pyrenophora teres f. teres	15A	NCBI	GCA_008086755.1	Pleosporaceae
Pyrenophora teres f. teres	6A	NCBI	GCA_008086725.1	Pleosporaceae
Pyrenophora teres f. teres	BB25	NCBI	GCA_008086785.1	Pleosporaceae
Pyrenophora teres f. teres	FGOH04Ptt-21	NCBI	GCA_008086845.1	Pleosporaceae
Pyrenophora teres f. teres	HRS9122	NCBI	GCA_009728645.1	Pleosporaceae
Pyrenophora teres f. teres	HRS9139	NCBI	GCA_009728635.1	Pleosporaceae
Pyrenophora teres f. teres	NB29	NCBI	GCA_009728665.1	Pleosporaceae
Pyrenophora teres f. teres	NB73	NCBI	GCA_009728655.1	Pleosporaceae
Pyrenophora teres f. teres	NB85	NCBI	GCA_009728675.1	Pleosporaceae
Pyrenophora teres f. teres	Pyrtt1	JGI	Pyrtt1	Pleosporaceae
Pyrenophora teres f. teres	W1-1	NCBI	GCA_900232045.2	Pleosporaceae
Pyrenophora tritici-repentis	134	NCBI	GCA_003231325.1	Pleosporaceae
Pyrenophora tritici-repentis	239	NCBI	GCA_003231365.1	Pleosporaceae
Pyrenophora tritici-repentis	5213	NCBI	GCA_003231345.1	Pleosporaceae
Pyrenophora tritici-repentis	11137	NCBI	GCA_003231355.1	Pleosporaceae
Pyrenophora tritici-repentis	86-124	NCBI	GCA_003231425.2	Pleosporaceae
Pyrenophora tritici-repentis	ARCrossB10	NCBI	GCA_018492725.1	Pleosporaceae
Pyrenophora tritici-repentis	DW5	NCBI	GCA_003231415.2	Pleosporaceae
Pyrenophora tritici-repentis	M4	NCBI	GCA_003171515.2	Pleosporaceae

Species	Strain	Source	Accession	Family
Pyrenophora tritici-repentis	Pt-1C-BFP	NCBI	GCA_000149985.1	Pleosporaceae
Pyrenophora tritici-repentis	Pyrtr1	JGI	Pyrtr1	Pleosporaceae
Pyrenophora tritici-repentis	V0001	NCBI	GCA_008692205.1	Pleosporaceae
Sclerotiophoma versabilis	KC1	NCBI	GCA_012274445.1	Didymellaceae
Setomelanomma holmii	CBS 110217	NCBI	GCA_010015745.1	Phaeosphaeriaceae
Shiraia sp.	Slf14	NCBI	GCA_000498155.1	Shiraiaceae
Sporormia fimetaria	CBS 119925	NCBI	GCA_010093795.1	Sporormiaceae
Stagonospora sp.	SRC1lsM3a	NCBI	GCA_001644525.1	Massarinaceae (Phaeosphaeriaceae?)
Stagonosporopsis cucurbitacearum	DBTL4	NCBI	GCA_016920805.1	Didymellaceae
Stagonosporopsis sp.	XDPOP-RS-16W	NCBI	GCA_016767195.1	Didymellaceae
Stagonosporopsis tanaceti	four strains pooled	NCBI	GCA_000812845.1	Didymellaceae
Stemphylium lycopersici	CIDEFI212	NCBI	GCA_003268315.1	Pleosporaceae
Stemphylium lycopersici	CIDEFI213	NCBI	GCA_003268335.1	Pleosporaceae
Stemphylium lycopersici	CIDEFI-216	JGI	Stely1	Pleosporaceae
Stemphylium vesicarium	173-1a-13FI1M3	NCBI	GCA_004380135.1	Pleosporaceae
Stemphylium vesicarium	On16-391	NCBI	GCA_008271585.1	Pleosporaceae
Stemphylium vesicarium	On16-63	NCBI	GCA_008271615.1	Pleosporaceae
Trematosphaeria pertusa	CBS 122368	NCBI	GCF_010094035.1	Trematosphaeriaceae
Westerdykella ornata	CBS 379.55	NCBI	GCF_010094085.1	Sporormiaceae
Westerdykella sp.	P71	NCBI	GCA_019022765.1	Sporormiaceae

10 List of fungal species for which the dehydrocurvularin biosynthetic genes were found from the whole-genome sequence database (281 Pleosporalean fungi).

Species	Strain	Percent identity with DET 2035 gene			
		dhc2	***dhc3***	***dhc4***	***dhc5***
Alternaria alternata	KAS5743	98.45	97.95	97.66	98.12
Alternaria arborescens	DET2035	100.00	100.00	100.00	100.00
Alternaria arborescens	KAS5321	99.95	99.91	99.96	99.83
Alternaria atra	CS162	98.66	98.46 (i)	98.22	98.46
Alternaria atra	MOD1-FUNGI7	98.56	98.51 (i)	98.17	98.47 (s)
Alternaria carthami	BMP1963	94.91	95.57	95.28	95.04
Alternaria consortialis	JCM1940	98.45	98.29	98.17	98.27
Alternaria macrospora	BMP1949	95.02	95.51	94.60	94.81
Alternaria porri	BMP0178	94.19 (*)	95.41	95.37	94.99
Alternaria solani	BMP0185	94.91	95.28	95.41	94.99
Alternaria solani	HWC-168-2012p	94.86	95.25	95.41	94.98
Alternaria solani	NL03003	94.91	95.25	95.41	94.99
Alternaria tomatophila	BMP2032	95.02 (*)	95.35 (s)	95.20	94.68 (s)
Pyrenophora tritici-repentis	134	89.76	87.52	87.34	88.23
Pyrenophora tritici-repentis	239	89.76	87.52	87.34	88.23
Pyrenophora tritici-repentis	5213	89.76	87.52	87.34	88.23
Pyrenophora tritici-repentis	11137	89.76	87.52	87.34	88.23
Pyrenophora tritici-repentis	86-124	89.76	87.52	87.34	88.23
Pyrenophora tritici-repentis	ARCrossB10	89.76	87.52	87.34	88.23
Pyrenophora tritici-repentis	DW5	89.76	87.52	87.34	88.23
Pyrenophora tritici-repentis	M4	89.76	87.55	87.34	88.23
Pyrenophora tritici-repentis	Pt-1C-BFP	89.76	87.52	87.34	88.20
Pyrenophora tritici-repentis	Pyrtr1	89.76	87.52	87.34	88.20
Pyrenophora tritici-repentis	V0001	89.76	87.52	87.34	88.22
Stemphylium vesicarium	173-1a-13FI1M3	98.23	97.92	97.62	98.19

(i) indicates an insertion occurs within the gene; (s) indicates the gene is split across multiple contigs; (*) indicates the entire gene is on a different contig than the other genes.
